# Correction: Health impact of monoclonal gammopathy of undetermined significance (MGUS) and monoclonal B-cell lymphocytosis (MBL): findings from a UK population-based cohort

**DOI:** 10.1136/bmjopen-2020-041296corr1

**Published:** 2022-07-06

Lamb MJ, Smith A, Painter D, *et al*. Health impact of monoclonal gammopathy of undetermined significance (MGUS) and monoclonal B-cell lymphocytosis (MBL): findings from a UK population-based cohort. *BMJ Open* 2021;11:e041296. doi: 10.1136/bmjopen-2020-041296

This article was previously published with an error in table 1. MGUS control numbers and percentages by gender were transposed and should read: males 11308 (51.6) and females 10620 (48.4).

