# Supplementary material for: Health impact of monoclonal gammopathy of undetermined significance (MGUS) and monoclonal B-cell lymphocytosis (MBL): findings from a UK population-based cohort
Source: BMJ Open. 2021 Feb 22;11(2):e041296. doi: 10.1136/bmjopen-2020-041296 (PMC7903106; doi:10.1136/bmjopen-2020-041296)
Supplement: Author's manuscript [file bmjopen-2020-041296.draft_revisions.pdf]

BMJ Open

BMJ Open is committed to open peer review. As part of this commitment we make the peer review history of every article we publish publicly available.

When an article is published we post the peer reviewers' comments and the authors' responses online. We also post the versions of the paper that were used during peer review. These are the versions that the peer review comments apply to.

The versions of the paper that follow are the versions that were submitted during the peer review process. They are not the versions of record or the final published versions. They should not be cited or distributed as the published version of this manuscript.

BMJ Open is an open access journal and the full, final, typeset and author-corrected version of record of the manuscript is available on our site with no access controls, subscription charges or pay-per-view fees (http://bmjopen.bmj.com).

If you have any questions on BMJ Open's open peer review process please email info.bmjopen@bmj.com

BMJ Open

Health impact of monoclonal gammopathy of undetermined significance (MGUS) and monoclonal B-cell lymphocytosis (MBL): findings from a UK population-based cohort

Journal:	*BMJ Open*
Manuscript ID	bmjopen-2020-041296
Article Type:	Original research
Date Submitted by the Author:	04-Jun-2020

Complete List of Authors:	Lamb, Maxine; University of York, Department of Health Sciences<br>Smith, Alexandra<br>Painter, Daniel<br>Kane, Eleanor<br>Bagguley, Timothy<br>Newton, Robert<br>Howell, Debra<br>Cook, Gordon<br>de Tute, Ruth<br>Rawstron, Andrew<br>Patmore, Russell<br>Roman, Eve

Keywords:	Myeloma < HAEMATOLOGY, Lymphoma < HAEMATOLOGY, EPIDEMIOLOGY

SCHOLARONE™
Manuscripts

I, the Submitting Author has the right to grant and does grant on behalf of all authors of the Work (as defined in the below author licence), an exclusive licence and/or a non-exclusive licence for contributions from authors who are: i) UK Crown employees; ii) where BMJ has agreed a CC-BY licence shall apply, and/or iii) in accordance with the terms applicable for US Federal Government officers or employees acting as part of their official duties; on a worldwide, perpetual, irrevocable, royalty-free basis to BMJ Publishing Group Ltd ("BMJ") its licensees and where the relevant Journal is co-owned by BMJ to the co-owners of the Journal, to publish the Work in this journal and any other BMJ products and to exploit all rights, as set out in our licence.

The Submitting Author accepts and understands that any supply made under these terms is made by BMJ to the Submitting Author unless you are acting as an employee on behalf of your employer or a postgraduate student of an affiliated institution which is paying any applicable article publishing charge ("APC") for Open Access articles. Where the Submitting Author wishes to make the Work available on an Open Access basis (and intends to pay the relevant APC), the terms of reuse of such Open Access shall be governed by a Creative Commons licence – details of these licences and which Creative Commons licence will apply to this Work are set out in our licence referred to above.

Other than as permitted in any relevant BMJ Author's Self Archiving Policies, I confirm this Work has not been accepted for publication elsewhere, is not being considered for publication elsewhere and does not duplicate material already published. I confirm all authors consent to publication of this Work and authorise the granting of this licence.

For peer review only

Health impact of monoclonal gammopathy of undetermined significance (MGUS) and monoclonal B-cell lymphocytosis (MBL): findings from a UK population-based cohort

Corresponding author: Eve Roman, Epidemiology & Cancer Statistics Group, Department of Health Sciences, Seebohm Rowntree Building, University of York, YO10 5DX, UK: eve.roman@york.ac.uk

Maxine Lamb[1], Alexandra G Smith[1], Daniel Painter[1], Eleanor Kane[1], Tim Bagguley[1], Robert Newton[1,2], Debra Howell[1], Gordon Cook[3], Ruth de Tute[4], Andrew Rawstron[4], Russell Patmore[5], Eve Roman[1]

[1] Department of Health Sciences, University of York, YO10 5DD, UK

[2] Ugandan Virus Research Institute, Entebbe, Uganda

[3] Department of Haematology, Leeds Cancer Centre, St James's University Hospital, LS9 7LP, UK

[4] Haematological Malignancy Diagnostic Service, Leeds Cancer Centre, St James's University Hospital, LS9 7LP, UK

[5] Queens Centre for Oncology and Haematology, Hull University Teaching Hospitals NHS Trust, HU16 5JQ, UK

For peer review only

For peer review only - http://bmjopen.bmj.com/site/about/guidelines.xhtml

ABSTRACT

Objective: To examine co-morbidity patterns in individuals with monoclonal gammopathy of undetermined significance (MGUS) and monoclonal B-cell lymphocytosis (MBL), both before and after premalignancy diagnosis; and compare their activity to that of the general population.

Design: Population-based patient cohort, within which each patient is matched at diagnosis to 10 age and sex-matched individuals from the general population. Both cohorts are linked to nationwide information on deaths, cancer registrations, and Hospital Episode Statistics (HES).

Setting: The UK's Haematological Malignancy Research Network; which has a catchment population of around 4 million served by 14 hospitals and a central diagnostic laboratory.

Participants: All patients newly diagnosed 2009-15 with MGUS (n=2203) or MBL (n=561), and their age and sex-matched comparators (n= 27,638).

Main Outcome measures: Survival, and hospital inpatient and outpatient activity in the five years before, and three years after, diagnosis.

Results: Individuals with MGUS experienced excess morbidity in the 5-years before diagnosis, and excess mortality and morbidity in the 3-years after. Increased rate ratios (RR) were evident for nearly all clinical specialties; the largest, both before and after diagnosis, being for nephrology (before RR=4.38, 95% Confidence Interval 3.99-4.81; after RR=14.7, 95% CI 13.5-15.9) and rheumatology (before RR=3.38, 95% CI 3.16-3.61; after RR=5.45, 95% CI 5.09-5.83). Strong effects were also evident for endocrinology, neurology, dermatology and respiratory medicine. Conversely, only marginal increases in mortality and morbidity were evident for MBL.

Conclusions: From a haematological malignancy perspective, MGUS and MBL are generally considered to be relatively benign. Nonetheless, monoclonal gammopathy has the potential to cause systemic disease and wide-ranging damage to most organs and tissues. Hence, even though most people with monoclonal immunoglobulins never develop a B-cell malignancy or suffer from any other form of M-protein related organ/tissue related disorder, the consequences for those that do can be extremely serious.

For peer review only

ARTICLE SUMMARY

Strengths and limitations of this study

- Data are from an established population-based cohort within which all haematological malignancies and related clonal disorders are diagnosed, monitored, and coded using up-to-date procedures at a central haematopathology laboratory.
- Providing nationally generalizable data, all diagnoses are included, and complete follow-up is achieved via linkage to nationwide information on mortality and morbidity.
- The age and sex-matched general population cohort enables baseline activity and rate ratios to be calculated, both before and after premalignancy detection.
- Most people with premalignant clonal disorders never suffer any ill-effects; and patients that are ascertained are often diagnosed as part of routine diagnostic work-up procedures.
- Analyses are constrained by the fact that hospital episodes statistics are primarily collected for administrative and clinical purposes, and not for research.

Funding

This work was supported by Cancer Research UK, grant numbers 18362 and 29685; and Blood Cancer UK, grant number 15037

Competing interests

None

Data sharing agreement

Ethical approvals and data restrictions mean that data cannot be shared; but collaborative projects can be undertaken. The corresponding author can be contacted for more information.

For peer review only - http://bmjopen.bmj.com/site/about/guidelines.xhtml

BACKGROUND

Monoclonal gammopathy of undetermined significance (MGUS) and monoclonal B-cell lymphocytosis (MBL) are premalignant monoclonal B-cell disorders; the former progressing to myeloma at a rate of around 1% per year[1,2] and the latter to chronic lymphocytic leukaemia (CLL) at around 2% per year.[3,4] Diagnosed more frequently in men than women and people over 60 years of age,[5,6] overt symptoms of haematological malignancy are, by definition, absent in both MBL and MGUS.[7] Accordingly, although some premalignant disorders are found coincidentally during routine health checks, others are identified during diagnostic work-up investigations; MGUS during the course of tests applied to detect a range of potential conditions and illnesses,[8,9] and MBL during episodes of unexplained lymphocytosis.[4,10]

In addition to the association with haematological malignancy, individuals with MGUS or MBL can experience higher than expected levels of mortality and morbidity that are independent of cancer.[4,8,11–16] Indeed, although most individuals with these disorders suffer no obvious ill effects, interest in their relationship with other co-morbidities has increased markedly in recent years; MBL largely in relation to its potential to impact on the immune response,[17] and MGUS due to the systemic organ and tissue damage that can be caused by monoclonal immunoglobins secreted by the abnormal B-cell clone.[18] Hitherto, however, most information about these associations has been derived either from case-control studies established to look at risk factors for disease development (e.g. family history of disease), additional tests applied to specific patient groups (e.g. patients with kidney disease), or cohort studies that track individuals with either MGUS/MBL forwards in time from their diagnosis.[5,13,18] However, despite the undoubted interest in the sequence of health events, as far as we are aware no systematic population-based investigations of the co-morbidity patterns that precede and succeed a diagnosis of either MGUS or MBL have been undertaken in the same cohort.

With a view to shedding light on the health events occurring before and after the diagnosis of MGUS and MBL, the present report uses data from an established UK population-based patient cohort of haematological malignancies and related disorders to examine the co-morbidity patterns of individuals with these premalignancy clonal disorders (MGUS=2203, MBL=561). To enable effect size quantification, these patterns are compared to the baseline activity of an individually age- and sex-matched (10 per patient) general population comparison cohort.

For peer review only

For peer review only - http://bmjopen.bmj.com/site/about/guidelines.xhtml

METHODS

Cases are from the Haematological Malignancy Research Network (HMRN; www.hmrn.org), a specialist UK registry established in 2004 to provide robust generalizable data to inform contemporary clinical practice and research across the country as a whole.[19,20] Set within a catchment population of around four million that is served by 14 hospitals and has a socioeconomic profile which is broadly representative of the UK as a whole, all haematological cancers and related conditions are diagnosed and coded by clinical specialists at a single integrated haematopathology laboratory, the Haematological Malignancy Diagnostic Service (www.hmds.info), using standardized diagnostic criteria and the latest WHO ICD-O-3 classification.[7] Specifically, in relation to the present report, MBL was defined by a peripheral blood monoclonal B-cell count <5 x10^9/L and MGUS by the detection of paraprotein in peripheral blood and/or neoplastic lymphoplasmacytic/plasma cells in the bone marrow.

To facilitate comparisons with the general population, HMRN also has a general population cohort; all patients diagnosed 2009-15 were individually matched on age and sex to 10 unaffected individuals from the same catchment population, each one of which was assigned a pseudo-diagnosis date corresponding to the date of diagnosis of their matched case.[21,22] HMRN operates under a legal basis that permits data to be collected directly from clinical records without explicit consent; and all individuals in the patient cohort and the comparison cohort are linked to nationwide information on deaths, cancer registrations and Hospital Episode Statistics (HES).

Using similar methods to those previously described,[21–23] associations with hospital inpatient activity (HES Admitted Patient Care; HES-APC) and outpatient activity (HES Outpatient; HES-OP) in the five years prior to diagnosis/pseudo-diagnosis through to the three years after diagnosis were investigated. HES inpatient data contains ICD-10 (International Statistical Classification of Diseases 10th revision) illness codes derived from discharge summaries[24]; and associations with these were examined in relation to the 17 specific conditions in the Charlson Comorbidity Index.[25–27] By contrast, HES outpatient data contains details about the type of outpatient attendance; the majority being linked to consultant specialty codes (e.g. ophthalmology, rheumatology etc.), with the remainder largely comprising routine follow-up/monitoring, nurse-led clinic attendances (e.g. anticoagulant clinics) and consultations with allied health professionals (e.g. podiatry).

This report includes all patients (cases) who were newly diagnosed with either MGUS (n=2203) or MBL (n=561) between 1st January 2009 and 31st December 2015 and their matched controls

For peer review only - http://bmjopen.bmj.com/site/about/guidelines.xhtml

(n=27,638); those diagnosed with a haematological cancer within six months of their MGUS/MBL diagnosis were considered ineligible. All cases and controls were followed-up for cancer registration and death until March 2017, and hospital activity (inpatient and outpatient) until March 2016. Additionally, progressions and/or transformations in cases were identified through HMDS up to March 2017. Data were summarised using standard methods. Overall survival, hospital activity and rate ratios (RRs) were calculated using time-to-event analyses. The Stata programme 'strel' was used to estimate relative survival (RS), using age- and sex-specific background mortality rates from national life tables.[28,29] All analyses were conducted using Stata 16.0.

Patient and public involvement (PPI)

PPI is integral to HMRN, and takes place via a dedicated partnership, overseen by a lay committee. Patients from the partnership are involved in identifying key research questions and participate in all our funding applications. Furthermore, patients and their relatives routinely take part in the dissemination of HMRN's findings, which also occurs via our lay website: www.yhhn.org

RESULTS

Characteristics of individuals with either MGUS or MBL are summarized alongside their corresponding controls in Table 1. Both MGUS and MBL were more commonly diagnosed in males (51.6% MGUS, 58.5% MBL) and individuals over the age of 70 years (median diagnostic age 73.3 years for MGUS and 72.1 years for MBL). At 90.0% (95% Confidence Interval 87.2-92.3%), the 5-year relative survival (RS) of patients diagnosed with MGUS was significantly lower ($p<0.01$) than that of their general population controls (RS 99.5%, 95% CI 98.7-99.8%). For MBL, the difference was far less marked; the 5-year RS being 94.3% (95% CI 86.3-97.7%) for MBL cases and 98.5% (95% CI 96.8-99.3%) for their controls. Interestingly, the observed variations in blood cancer progression were in the opposite direction to those seen for mortality: 75 (3.4%) MGUS patients were diagnosed with a haematological malignancy (48/75 were myelomas) before April 2017, compared to 140 (25.0%) MBL patients (137/140 were CLLs). No significant differences were, however, evident for non-haematological cancer registrations; although the frequencies were slightly higher for MGUS (8.4%) and MBL (10.3%) than for their corresponding controls (8.0% and 8.8% respectively).

For peer review only - http://bmjopen.bmj.com/site/about/guidelines.xhtml

Table 1: Characteristics of patients diagnosed with monoclonal gammopathy of undetermined significance (MGUS) or monoclonal B-cell lymphocytosis (MBL) and their corresponding controls; HMRN diagnoses 2009-2015

		MGUS		MBL	
		Cases Number (%)	Controls Number (%)	Cases Number (%)	Controls Number (%)
**Total**		2203 (100)	22028 (100)	561 (100)	5610 (100)
**Gender**	**Male**	1136 (51.6)	11358 (51.6)	328 (58.5)	3280 (58.5)
	**Female**	1067 (48.4)	10670 (48.4)	233 (41.5)	2330 (41.5)
**Age (years)**	**<60**	349 (15.8)	3490 (15.8)	85 (15.2)	850 (15.2)
	**60-70**	502 (22.8)	5020 (22.8)	153 (27.3)	1530 (27.3)
	**70-80**	788 (35.8)	7880 (35.8)	194 (34.6)	1940 (34.6)
	**≥80**	564 (25.6)	5638 (25.6)	129 (23.0)	1290 (23.0)
	**Median (IQR)**	73.3 (64.6 - 80.2)	73.3 (64.6 - 80.2)	72.1 (64.5 - 79.2)	72.1 (64.5 - 79.2)
**Overall survival[1]**	**5-year (95% CI)**	71.8 (69.5-74.0)	80.1 (79.5-80.8)	77.2 (72.5-81.2)	80.7 (79.4-81.9)
**Relative survival**	**5-year (95% CI)**	90.0 (87.2-92.3)	99.5 (98.7-99.8)	94.3 (86.3, 97.7)	98.5 (96.8-99.3)
**Progressions and Transformations[1]**	**Total**	75 (3.4)		140 (25.0)	
	**Myeloma**	48 (2.2)	-	-	-
	**CLL[2]**	1 (0.05)	-	137 (24.4)	-
	**Other**	26 (1.2)	-	3 (0.5)	-
**Non-haematological cancer registrations[1]**	**Total**	186 (8.4)	1759 (8.0)	58 (10.3)	494 (8.8)
	**Lung**	32 (1.5)	191 (0.9)	4 (0.7)	59 (1.1)
	**Prostate[3]**	20 (1.8)	179 (1.6)	3 (0.9)	56 (1.7)
	**Colorectal**	19 (0.9)	167 (0.8)	6 (1.1)	40 (0.7)
	**Breast[3]**	15 (1.4)	135 (1.3)	4 (1.7)	16 (0.7)
**Comorbidity score before MGUS/MBL diagnosis[4]**	**0**	1262 (57.3)	15975 (72.5)	419 (74.7)	4115 (73.4)
	**1**	510 (23.2)	3630 (16.5)	84 (15.0)	898 (16.0)
	**≥2**	431 (19.6)	2423 (11.0)	58 (10.3)	597 (10.6)
		p<0.001		p=0.78	

[1]Followed-up until 15/03/2017; [2]Chronic lymphocytic leukaemia; [3]Prostate for men only, breast for women only; [4]≥1 hospital admission with a diagnosis of one the 17 conditions included in the Charlson Comorbidity Index[25-27] during the 5 years to 1 month before MGUS/MBL diagnosis (patients)/pseudo-diagnosis (controls)

With respect to co-morbidity, in the years leading up to diagnosis MGUS patients were significantly more likely than their corresponding controls to have a record of at least one of the 17 co-morbidities specified in the Charlson Comorbidity Index[25–27] recorded in their discharge summaries; but no difference between MBL cases and their controls was evident (Table 1). More information about the extent of the differences between the hospital activity patterns of cases with MGUS/MBL and their general population controls, is shown in Figure 1; which shows inpatient and

For peer review only - http://bmjopen.bmj.com/site/about/guidelines.xhtml

For peer review only

outpatient activity (excluding haematology) during the five-years before and the three-years after diagnosis of MGUS (Figure 1A) and MBL (Figure 1B). In the period before diagnosis, patients with MGUS (Figure 1A) had consistently higher outpatient activity rates than their controls, the disparity increasing markedly during the 18 months leading up to the formal diagnosis of MGUS by haematopathology. Although less pronounced, a similar pattern is evident in inpatient data. With smaller numbers and more scatter, variations in outpatient and inpatient activity in MBL are less evident (Figure 1B).

Figure 2 shows outpatient attendance frequencies (at least two specialty-specific visits) in the three years before and in the three years after MGUS diagnosis for the top 25 clinical specialties; visits within one month (±) of diagnosis/pseudodiagnosis are excluded. As is evident from the plot, the increased outpatient activity seen among cases (Figure 1) occurs across a range of clinical specialties; the highest frequencies occurring in ophthalmology, haematology, general surgery, orthopaedics, general (internal) medicine and rheumatology. However, excluding haematology where, as expected, attendances increased markedly just before and after MGUS diagnosis, the largest rate ratios (RR) both before (Figure 3A) and after (Figures 3B) diagnosis were for nephrology (before diagnosis RR= 4.38, 95% CI 3.99-4.81; after diagnosis RR= 14.7, 95% CI 13.5-15.9) and rheumatology (before diagnosis RR= 3.38, 95% CI 3.16-3.61; after diagnosis RR= 5.45, 95% CI 5.09-5.83). Other significant associations ($p<0.05$) with rate ratio point estimates above 2.0 were evident for endocrinology, neurology, and respiratory medicine, as well as for the nurse-led monitoring activities which form part of on-going clinical care across a range of specialties.

MGUS data are stratified by subtype in Table 2. Accounting for around two-thirds of the total (n=1469; 66.7%) the IgG subtype dominates, followed by IgM (n=349; 15.8%) and IgA (n=268; 12.2%). The remaining 117 (5.3%) 'other' category comprise a mix of subtypes: light chain only (n=62), IgG + IgM (n=17), IgG + IgA (n=6), IgA + IgM (n=1), IgE (n=2), IgD (n=1); and not recorded (n=28). As expected, progression to myeloma in the three years following MGUS diagnosis was largely restricted to the IgG and IgA subtypes. The age distributions, five-year survival estimates (overall and relative) and non-haematological malignancy frequencies of the main subtypes were broadly similar; although patients in the combined 'other' category tended to be slightly older and to fare less well (RS= 75.1%, 95% CI 60.8-84.9%). The numbers of patients in the individual groups were, however, too sparse to examine the data in greater depth.

For peer review only - http://bmjopen.bmj.com/site/about/guidelines.xhtml

Table 2: Characteristics of patients diagnosed with monoclonal gammopathy of undetermined significance (MGUS) and their corresponding controls, stratified by MGUS subtype; HMRN diagnoses 2009-2015

	MGUS subtype							
	IgG		IgM		IgA		Other[1]	
	Cases Number (%)	Controls Number (%)	Cases Number (%)	Controls Number (%)	Cases Number (%)	Controls Number (%)	Cases Number (%)	Controls Number (%)
**Total**	1469 (100.0)	14689 (100.0)	349 (100.0)	3489 (100.0	268 (100.0)	2680 (100.0)	117 (100.0)	1170 (100.0)
**Gender**								
**Male**	741 (50.4)	7409 (50.4)	177 (50.9)	1769 (50.7)	146 (54.5)	1460 (54.5)	72 (61.5)	720 (61.5)
**Female**	728 (49.6)	7280 (49.6)	172 (49.3)	1720 (49.3)	122 (45.5)	1220 (45.5)	45 (38.5)	450 (38.5)
**Age (years)**								
**<60**	241 (16.4)	2410 (16.4)	50 (14.3)	500 (14.3)	48 (17.9)	480 (17.9)	10 (8.5)	100 (8.5)
**60-70**	335 (22.8)	3350 (22.8)	79 (22.6)	790 (22.6)	56 (20.9)	560 (20.9)	32 (27.4)	320 (27.4)
**70-80**	513 (34.9)	5130 (34.9)	130 (37.2)	1300 (37.3)	106 (39.6)	1060 (39.6)	39 (33.3)	390 (33.3)
**≥80**	380 (25.9)	3799 (25.9)	90 (25.8)	899 (25.8)	58 (21.6)	580 (21.6)	36 (30.8)	360 (30.8)
**Median**	73.4	73.4	73.1	73.1	73.0	73.0	74.5	74.5
**Survival 5-year (95% CI)[2]**								
**Overall**	72.3 (69.5, 75.0)	79.5 (78.7, 80.3)	69.5 (63.3, 75.0)	81.3 (79.7, 82.8)	74.4 (67.5, 80.0)	82.5 (80.6, 84.1)	65.8 (54.1, 75.1)	78.6 (75.3, 81.5)
**Relative**	90.6 (87.0, 93.2)	99.2 (98.2, 99.6)	84.1 (75.8, 89.8)	99.3 (93.8, 99.9)	88.5 (78.7, 93.9)	98.5 (95.4, 99.5)	75.1 (60.6, 84.9)	97.2 (90.3, 99.2)
**Progressions/transformations[2]**								
**Total**	43 (2.9)	-	8 (2.3)	-	17 (6.3)	-	7 (6.0)	-
**Myeloma**	33 (2.2)	-	1 (0.3)	-	11 (4.1)	-	3 (2.6)	-
**Other**	10 (0.7)	-	7 (2.0)	-	6 (2.2)	-	4 (3.4)	-
**Non-haematological cancer registrations[2]**								
**Total**	115 (7.8)	1175 (8.0)	41 (11.7)	277 (7.9)	20 (7.5)	212 (7.9)	10 (8.5)	95 (8.1)
**Lung**	16 (1.1)	134 (0.9)	6 (1.7)	24 (0.7)	4 1.5)	21 (0.8)	6 (5.1)	12 (1.0)
**Prostate[3]**	12 (1.6)	114 (1.5)	3 (1.7)	27 (1.5)	3 (2.1)	25 (1.7)	2 (2.8)	13 (1.8)
**Colorectal**	16 (1.1)	113 (0.8)	2 (0.6)	26 (0.7)	1 (0.4)	20 (0.7)	-	8 (0.7)
**Breast[3]**	9 (1.2)	88 (1.2)	4 (2.3)	26 (1.5)	2 (1.6)	15 (1.2)	-	6 (1.3)
**Hospital outpatient activity (≥ 3 visits), excluding haematology and visits within 1 month of diagnosis[4]**								
**5-years before diagnosis**	1171 (79.7)	9086 (61.9)	286 (81.9)	2149 (61.6)	218 (81.3)	1645 (61.4)	99 (84.6)	728 (62.2)
**3-years after diagnosis**	966 (65.8)	6547 (44.6)	240 (68.8)	1575 (45.1)	178 (66.4)	1165 (43.5)	76 (65.0)	495 (42.3)

[1]Biclonal, IgD, IgE, light chain, not known; [2]Followed-up until 15/03/2017; [3]Prostate for men only, breast for women only; [4]In the 5 years before diagnosis/pseudo-diagnosis and 3 years after diagnosis/pseudo-diagnosis

For peer review only - http://bmjopen.bmj.com/site/about/guidelines.xhtml

DISCUSSION

Including data on nearly 3000 cases with pre-malignant clonal disorders and ten times as many age and sex-matched general population controls, this large UK record-linkage study found that individuals with MGUS not only experienced excess mortality and morbidity after diagnosis, but also excess morbidity in the 5 years before. By contrast, only marginal increases in mortality and morbidity were evident for MBL; none of which were consistent or varied significantly from the general population. Interestingly, progression patterns were in the opposite direction: in the years following detection of a pre-malignant clonal disorder, 3.4% (n=75/2203) of those with MGUS developed a haematological malignancy (41 of which were myelomas) before April 2017, compared to 25.0% (n=140/561) of those with MBL (137 of which were CLLs).

The elevated mortality and morbidity following a diagnosis of MGUS, which seems largely independent of progression to cancer, is consistent with reports relating to the potential clinical significance of this disorder.[11–18] In addition, our findings not only demonstrate the range and strength of the morbidity effects, but also show that levels are elevated many years before MGUS is diagnosed: excesses being observed in specialties covering most organ and tissue systems including nephrology, endocrinology, neurology, rheumatology, gastroenterology, dermatology and respiratory medicine. With respect to MBL, even though a quarter of patients developed CLL during the follow-up period, no effect on survival was detected. This result is, however, similar to that reported by other studies that used age and sex-matched controls.[3,30] Furthermore, although hospital activity increased in the months around the time of MBL diagnosis, no consistent differences or patterns before or after then, were detected. The number of patients in our MBL cohort (n=561), although comparatively large is nonetheless smaller than the number of patients in our MGUS cohort (n=2203). Furthermore, given the fact that MBL has been associated with increased susceptibility to infection and non-CLL related mortality[4,15,31], it is possible that the findings relating to subsequent morbidity and mortality could change as our data mature, length of follow-up increases, and linkage to primary care data occurs.

The age and sex distributions of our population-based cohorts are broadly similar to those of other published MBL[3,32,33] and MGUS[1,2,11] series; as is the dominance of the IgG MGUS subtype.[1,2] Providing nationally generalizable data, additional strengths of our study include its large well-defined population, within which all haematological malignancies and related clonal disorders are diagnosed, monitored, and coded using up-to-date standardized procedures at a central haematopathology laboratory.[19] In this context, it is important to bear in mind that most people with premalignant clonal disorders remain asymptomatic, and that our cohorts contain a relatively large proportion of people who came to clinical attention in primary and/or secondary care. Hence, our patient cohorts are likely to represent the more severe end of the disease spectrum; prevalence comparisons with population screening studies suggesting that this could be as low as 5% of the those ≥50 years with monoclonal immunoglobulin

For peer review only

in their blood/urine.[5,20,33–35] Furthermore, during the study period (2009-15), in addition to the detection of a paraprotein in peripheral blood, around 70% (1546/2203) of MGUS patients in our cohort had a confirmatory bone marrow sample. The patient characteristics and secondary care activity patterns of those who had a bone marrow sample were, however, broadly similar to those who did not (data not shown).

The diversity of morbidity effects seen among individuals with MGUS is consistent with the expanding body of evidence relating to the potential adverse impact that even low levels of circulating monoclonal protein (M-protein) can have. Thus far, the complex underpinning mechanisms identified include; deposition of M-protein aggregations of varying immunoglobulin subtypes in different organs, as well as the induction of auto-antibodies and cytokines that can impact on organs and tissues in a variety of deleterious ways.[13,18,36,37] Indeed, the recognized number of M-protein mediated entities is increasing, with several affecting multiple organs; well-known deposition syndromes including primary amyloidosis and paraneoplastic conditions such as POEMS syndrome (polyneuropathy, organomegaly, endocrinopathy, monoclonal gammopathy, and skin changes).[7,38] As evidenced in our analysis, kidney involvement is frequent, both in the years before (four-fold excess) and after (fifteen-fold excess) MGUS diagnosis. Indeed, the umbrella term monoclonal gammopathy of renal significance (MGRS) has recently been suggested to cover all M-protein mediated kidney disorders that fail to meet the diagnostic criteria for multiple myeloma or any other B-cell malignancy.[13,18,39] Other organ-specific terms continue to emerge, and with a view to improving recognition of these complex disorders which clearly pose significant diagnostic and treatment challenges, the overarching term monoclonal gammopathy of clinical significance (MGCS) has also been suggested.[18]

From a haematological malignancy perspective, MGUS and MBL are generally considered to be relatively benign conditions. However, both can have other deleterious health consequences; the effect of monoclonal gammopathy being particularly striking. Impacting significantly on survival and having the potential to cause systemic disease and wide-ranging damage to most organs and tissues, the adverse outcomes associated with the M-proteins produced by the abnormal B-cell clone can be severe and extend over many years. Even though most people with monoclonal immunoglobulins never develop a B-cell malignancy or suffer from any other form of M-protein related organ/tissue related disorder, the consequences for those that do can be extremely serious. In this regard, early targeting of pathogenic B-cell clones could mitigate both cancer and non-cancer effects; but currently, although knowledge is increasing, there is no known way to reliably identify such clones in the absence of other signs/symptoms. Hence, population screening cannot be recommended, and diagnosis remains reliant on clinical suspicion. However, the long-standing nature of the co-morbidity associations seen prior to MGUS diagnosis in our data, suggest that there may be room for improvement. Hence, in the future, the implementation of

For peer review only

strategies to improve awareness and earlier detection, as well as monitoring of high-risk patient groups, could prove beneficial.

Ethics Approval: The Haematological Malignancy Research Network has ethics approval (REC 04/01205/69) from Leeds West Ethics Committee, R&D approval from each NHS Trust, and exemption from Section 251 of the Health & Social Care Act (PIAG 1-05 9h)/2007)

Contributors: ER, AS, DH and RP initiated the patient cohort within which this research is nested; and ER, AS, and EK designed the comparison cohort. ER, AS and ML planned this study and analyses. R de T and AR oversaw all laboratory procedures; and ML, DP, EK, TB managed the data and carried out data analysis. GC, RP, AR, and RN commented on the biological and clinical aspects. ER and ML drafted the manuscript, which was approved by all authors.

References

1 Kyle RA, Larson DR, Therneau TM, *et al.* Long-Term Follow-up of Monoclonal Gammopathy of Undetermined Significance. *N Engl J Med* 2018;**378**:241–9. doi:10.1056/NEJMoa1709974

2 Turesson I, Kovalchik SA, Pfeiffer RM, *et al.* Monoclonal gammopathy of undetermined significance and risk of lymphoid and myeloid malignancies: 728 cases followed up to 30 years in Sweden. *Blood* 2014;**123**:338–45. doi:10.1182/blood-2013-05-505487

3 Parikh SA, Chaffee KG, Larson MC, *et al.* Outcomes of a large cohort of individuals with clinically ascertained high-count monoclonal B-cell lymphocytosis. *Haematologica* 2018;**103**:e237–40. doi:10.3324/haematol.2017.183194

4 Rawstron AC, Bennett FL, O'Connor SJM, *et al.* Monoclonal B-cell lymphocytosis and chronic lymphocytic leukemia. *N Engl J Med* 2008;**359**:575–83. doi:10.1056/NEJMoa075290

5 Goldin LR, McMaster ML, Caporaso NE. Precursors to lymphoproliferative malignancies. *Cancer Epidemiol Biomarkers Prev* 2013;**22**:533–9. doi:10.1158/1055-9965.EPI-12-1348

6 Smith A, Howell D, Patmore R, *et al.* Incidence of haematological malignancy by sub-type: a report from the Haematological Malignancy Research Network. *Br J Cancer* 2011;**105**:1684–92. doi:10.1038/bjc.2011.450

7 Swerdlow S, Campo E, Harris N, *et al.* *WHO Classification of Tumours of Haematopoietic and Lymphoid Tissues*. Lyon, France: : World Health Organization 2017.

8 Kristinsson SY, Björkholm M, Andersson TM-L, *et al.* Patterns of survival and causes of death following a diagnosis of monoclonal gammopathy of undetermined significance: a population-based study. *Haematologica* 2009;**94**:1714–20. doi:10.3324/haematol.2009.010066

9 Merlini G, Palladini G. Differential diagnosis of monoclonal gammopathy of undetermined significance. *Hematology Am Soc Hematol Educ Program* 2012;**2012**:595–603. doi:10.1182/asheducation.V2012.1.595.3798563

For peer review only

10 Shanafelt TD, Ghia P, Lanasa MC, *et al.* Monoclonal B-cell lymphocytosis (MBL): biology, natural history and clinical management. *Leukemia* 2010;**24**:512–20. doi:10.1038/leu.2009.287

11 Kristinsson SY, Tang M, Pfeiffer RM, *et al.* Monoclonal gammopathy of undetermined significance and risk of infections: a population-based study. *Haematologica* 2012;**97**:854–8. doi:10.3324/haematol.2011.054015

12 Thorsteinsdottir S, Lund SH, Lindqvist EK, *et al.* Bone disease in monoclonal gammopathy of undetermined significance: results from a screened population-based study. *Blood Adv* 2017;**1**:2790–8. doi:10.1182/bloodadvances.2017010454

13 Glavey SV, Leung N. Monoclonal gammopathy: The good, the bad and the ugly. *Blood Rev* 2016;**30**:223–31. doi:10.1016/j.blre.2015.12.001

14 Ciocchini M, Arbelbide J, Musso CG. Monoclonal gammopathy of renal significance (MGRS): the characteristics and significance of a new meta-entity. *Int Urol Nephrol* 2017;**49**:2171–5. doi:10.1007/s11255-017-1594-y

15 Moreira J, Rabe KG, Cerhan JR, *et al.* Infectious complications among individuals with clinical monoclonal B-cell lymphocytosis (MBL): a cohort study of newly diagnosed cases compared to controls. *Leukemia* 2013;**27**:136–41. doi:10.1038/leu.2012.187

16 Rögnvaldsson S, Steingrímsson V, Turesson I, *et al.* Peripheral neuropathy and monoclonal gammopathy of undetermined significance: a population-based study including 15,351 cases and 58,619 matched controls. *Haematologica* Published Online First: 13 February 2020. doi:10.3324/haematol.2019.239632

17 Criado I, Blanco E, Rodríguez-Caballero A, *et al.* Residual normal B-cell profiles in monoclonal B-cell lymphocytosis versus chronic lymphocytic leukemia. *Leukemia* 2018;**32**:2701–5. doi:10.1038/s41375-018-0164-3

18 Fermand J-P, Bridoux F, Dispenzieri A, *et al.* Monoclonal gammopathy of clinical significance: a novel concept with therapeutic implications. *Blood* 2018;**132**:1478–85. doi:10.1182/blood-2018-04-839480

19 Smith A, Howell D, Crouch S, *et al.* Cohort Profile: The Haematological Malignancy Research Network (HMRN); a UK population-based patient cohort. *Int J Epidemiol* Published Online First: 2018. doi:10.1093/ije/dyy044

20 Haematological Malignancy Research Network. HMRN - Statistics. https://www.hmrn.org/statistics (accessed 12 Apr 2020).

21 Kane E, Painter D, Smith A, *et al.* The impact of rheumatological disorders on lymphomas and myeloma: a report on risk and survival from the UK's population-based Haematological Malignancy Research Network. *Cancer Epidemiology* 2019;**59**:236–43. doi:10.1016/j.canep.2019.02.014

22 Kane E, Painter D, Smith A, *et al.* Risk of mature B-cell neoplasms and precursor conditions after joint replacement: a report from the Haematological Malignancy Research Network. *Int J Cancer* Published Online First: 1 November 2019. doi:10.1002/ijc.32765

23 Lamb MJ, Roman E, Howell DA, *et al.* Hodgkin lymphoma detection and survival: findings from the Haematological Malignancy Research Network. *BJGP Open* 2019;**3**. doi:10.3399/bjgpopen19X101668

24 World Health Organization. International Statistical Classification of Diseases and Related Health Problems, 10th revision. 2010.

For peer review only

25 Maringe C, Fowler H, Rachet B, *et al.* Reproducibility, reliability and validity of population-based administrative health data for the assessment of cancer non-related comorbidities. *PLOS ONE* 2017;**12**:e0172814. doi:10.1371/journal.pone.0172814

26 Wästerlid T, Mohammadi M, Smedby KE, *et al.* Impact of comorbidity on disease characteristics, treatment intent and outcome in diffuse large B-cell lymphoma: a Swedish lymphoma register study. *Journal of Internal Medicine* 2019;**285**:455–68. doi:10.1111/joim.12849

27 Charlson ME, Pompei P, Ales KL, *et al.* A new method of classifying prognostic comorbidity in longitudinal studies: Development and validation. *Journal of Chronic Diseases* 1987;**40**:373–83. doi:10.1016/0021-9681(87)90171-8

28 Estève J, Benhamou E, Croasdale M, *et al.* Relative survival and the estimation of net survival: Elements for further discussion. *Statist Med* 1990;**9**:529–38. doi:10.1002/sim.4780090506

29 Cancer Research UK Cancer Survival Group. UK life tables. UK life tables. 2017.http://csg.lshtm.ac.uk/tools-analysis/uk-life-tables/ (accessed 26 Jan 2018).

30 Strati P, Shanafelt TD. Monoclonal B-cell lymphocytosis and early-stage chronic lymphocytic leukemia: diagnosis, natural history, and risk stratification. *Blood* 2015;**126**:454–62. doi:10.1182/blood-2015-02-585059

31 Criado I, Rodríguez-Caballero A, Gutiérrez ML, *et al.* Low-count monoclonal B-cell lymphocytosis persists after seven years of follow up and is associated with a poorer outcome. *Haematologica* 2018;**103**:1198–208. doi:10.3324/haematol.2017.183954

32 Rawstron AC, Shanafelt T, Lanasa MC, *et al.* Different biology and clinical outcome according to the absolute numbers of clonal B-cells in monoclonal B-cell lymphocytosis (MBL). *Cytometry B Clin Cytom* 2010;**78 Suppl 1**:S19-23. doi:10.1002/cyto.b.20533

33 Call TG, Norman AD, Hanson CA, *et al.* Incidence of chronic lymphocytic leukemia and high-count monoclonal B-cell lymphocytosis using the 2008 guidelines. *Cancer* 2014;**120**:2000–5. doi:10.1002/cncr.28690

34 Wadhera RK, Rajkumar SV. Prevalence of Monoclonal Gammopathy of Undetermined Significance: A Systematic Review. *Mayo Clinic Proceedings* 2010;**85**:933–42. doi:10.4065/mcp.2010.0337

35 Li J, Smith A, Crouch S, *et al.* Estimating the prevalence of hematological malignancies and precursor conditions using data from Haematological Malignancy Research Network (HMRN). *Cancer Causes Control* 2016;**27**:1019–26. doi:10.1007/s10552-016-0780-z

36 Merlini G, Stone MJ. Dangerous small B-cell clones. *Blood* 2006;**108**:2520–30. doi:10.1182/blood-2006-03-001164

37 Atkin C, Richter A, Sapey E. What is the significance of monoclonal gammopathy of undetermined significance? *Clin Med (Lond)* 2018;**18**:391–6. doi:10.7861/clinmedicine.18-5-391

38 Dispenzieri A. POEMS Syndrome: 2019 Update on diagnosis, risk-stratification, and management. *Am J Hematol* 2019;**94**:812–27. doi:10.1002/ajh.25495

39 Amaador K, Peeters H, Minnema MC, *et al.* Monoclonal gammopathy of renal significance (MGRS) histopathologic classification, diagnostic workup, and therapeutic options. *Neth J Med* 2019;**77**:243–54.

For peer review only

Figure 1: Inpatient (IP) and outpatient (OP) monthly hospital visit activity rates five years before to three years after diagnosis (cases)/pseudo-diagnosis (controls) excluding haematology attendances: A) monoclonal gammopathy of undetermined significance (MGUS) and B) monoclonal B-cell lymphocytosis (MBL) diagnosed 2009-15

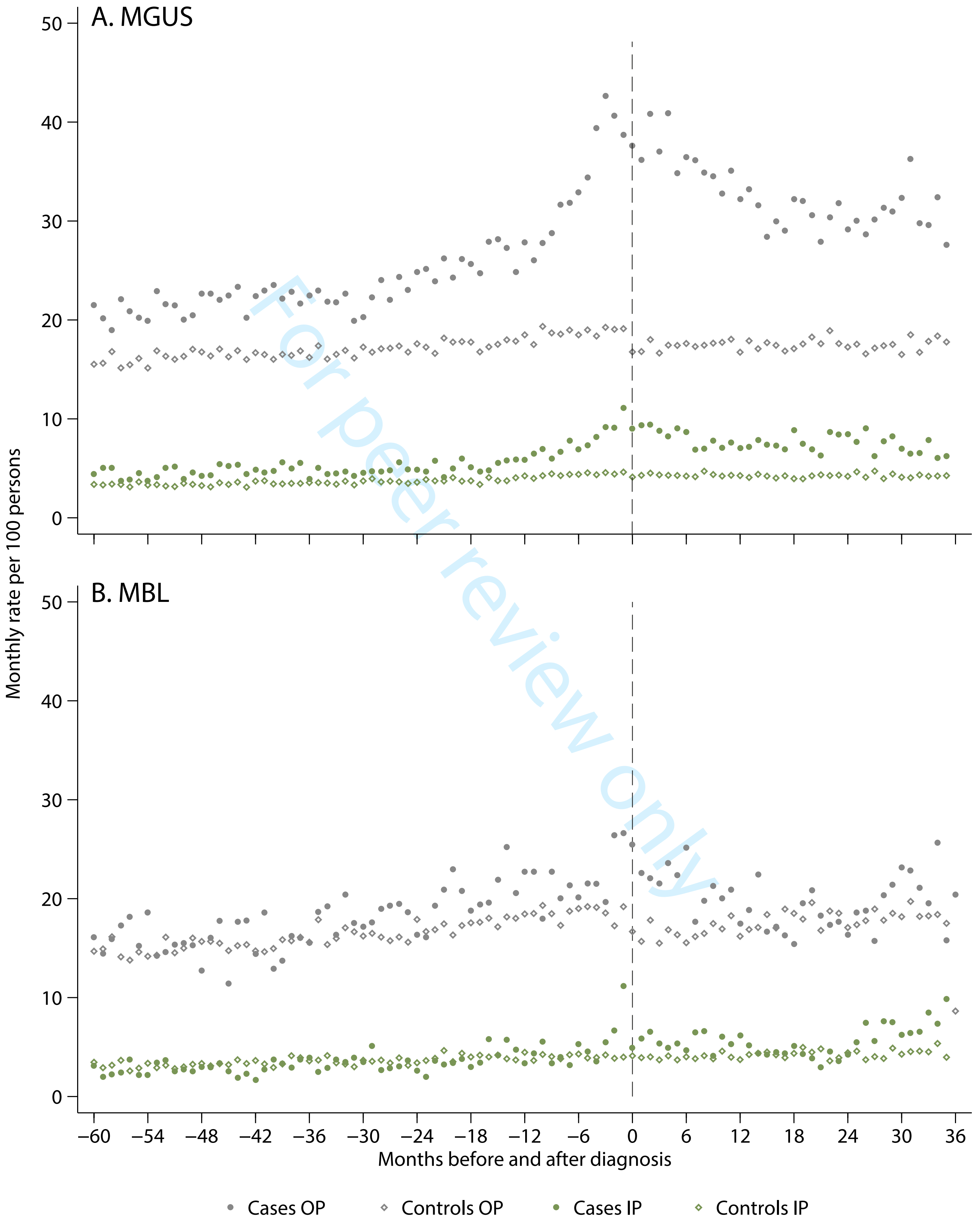

For peer review only

Figure 2: Percentage of cases and controls with at least two specialty-specific outpatient visits in the three years before and after diagnosis of monoclonal gammopathy of undetermined significance (MGUS). Top 25 recorded specialties, with visits within one month of diagnosis/pseudodiagnosis excluded.

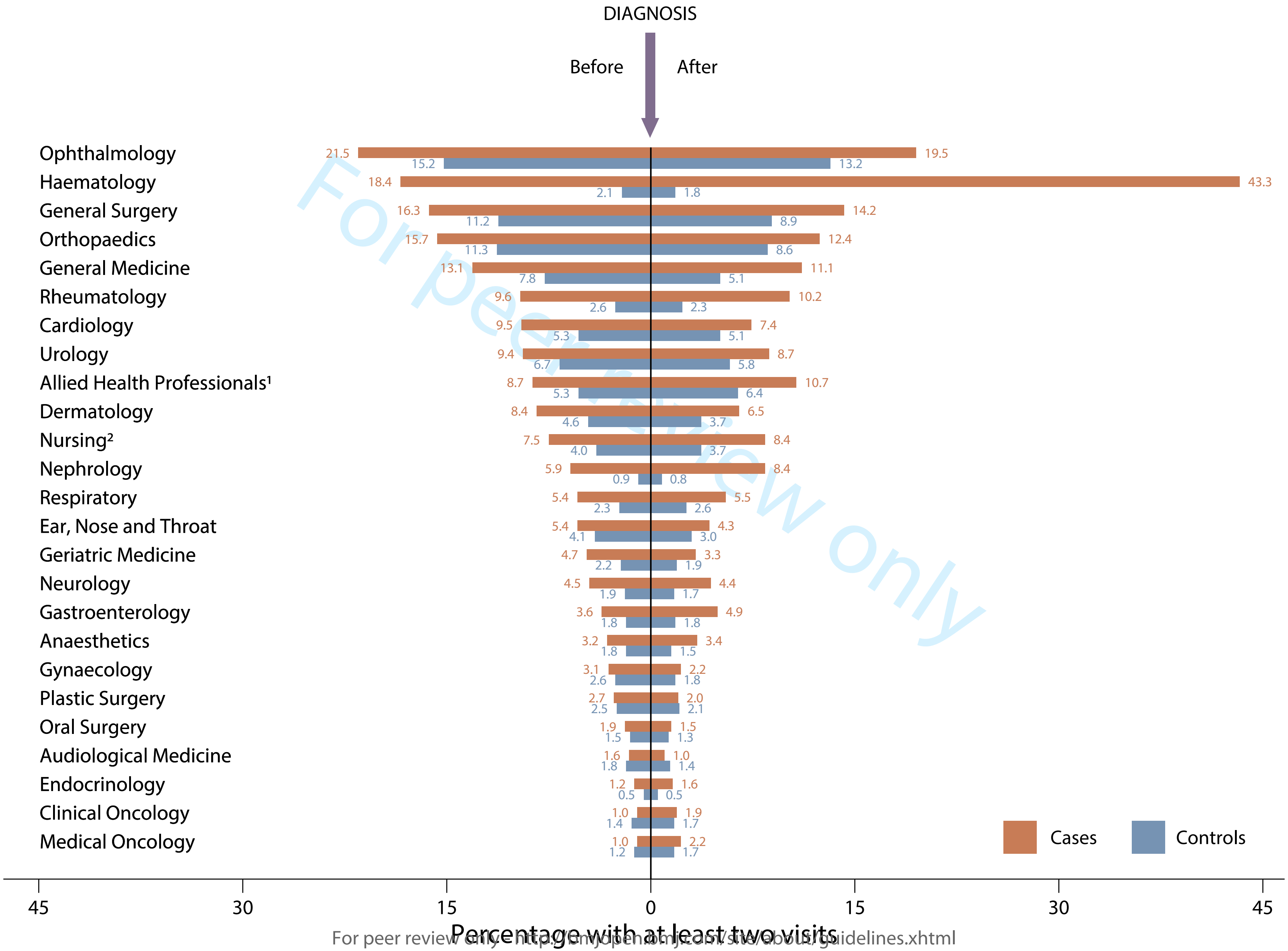

Figure 3: Monthly outpatient attendance rates (per 100 persons) in cases and controls and rate ratios by outpatient specialty with at least two visits (A) in the three years before diagnosis and (B) in the three years after diagnosis of monoclonal gammopathy of undetermined significance (MGUS)

Outpatient specialty	Post–diagnosis rank	Case rate	Control rate	Rate ratio (95% CI)
Nephrology	1	0.93	0.21	4.38 (3.99–4.81)
Rheumatology	2	1.69	0.50	3.38 (3.16–3.61)
Gastroenterology	6	1.46	0.62	2.35 (2.19–2.52)
Endocrinology	4	0.15	0.07	2.18 (1.76–2.69)
Neurology	8	0.75	0.35	2.14 (1.94–2.35)
Nursing	3	0.40	0.21	1.88 (1.66–2.14)
General Medicine	5	0.51	0.28	1.83 (1.63–2.05)
Respiratory	10	1.54	0.85	1.81 (1.70–1.94)
Anaesthetics	7	2.57	1.48	1.76 (1.66–1.86)
Dermatology	11	1.53	0.93	1.64 (1.54–1.75)
Allied Health Professionals	12	0.45	0.28	1.63 (1.45–1.84)
Geriatric Medicine	13	1.17	0.73	1.59 (1.48–1.72)
Cardiology	9	0.40	0.26	1.51 (1.33–1.71)
General Surgery	16	3.77	2.91	1.30 (1.24–1.35)
Orthopaedics	14	2.00	1.57	1.28 (1.21–1.35)
Ophthalmology	15	2.19	1.74	1.26 (1.19–1.32)
Ear, Nose and Throat	18	1.27	1.08	1.18 (1.10–1.26)
Urology	17	0.66	0.58	1.13 (1.03–1.24)
Gynaecology	20	0.42	0.40	1.05 (0.93–1.18)
Plastic Surgery	19	0.39	0.37	1.03 (0.91–1.17)

Outpatient specialty	Pre–diagnosis rank	Case rate	Control rate	Rate ratio (95% CI)
Nephrology	1	2.55	0.17	14.7 (13.5–15.9)
Rheumatology	2	2.08	0.39	5.45 (5.09–5.83)
Gastroenterology	6	0.61	0.20	3.00 (2.67–3.37)
Endocrinology	4	0.19	0.06	2.94 (2.37–3.63)
Neurology	7	0.66	0.25	2.67 (2.39–2.99)
Nursing	3	1.31	0.59	2.67 (2.48–2.86)
General Medicine	9	2.58	1.07	2.59 (2.44–2.75)
Respiratory	5	0.89	0.39	2.29 (2.08–2.52)
Anaesthetics	13	0.50	0.22	2.28 (2.01–2.58)
Dermatology	8	1.53	0.71	2.16 (2.01–2.32)
Allied Health Professionals	10	2.13	1.14	1.87 (1.76–1.99)
Geriatric Medicine	11	0.40	0.22	1.84 (1.60–2.11)
Cardiology	12	1.15	0.69	1.67 (1.54–1.81)
General Surgery	15	2.08	1.27	1.64 (1.54–1.74)
Orthopaedics	16	2.15	1.40	1.54 (1.45–1.63)
Ophthalmology	14	3.88	2.57	1.51 (1.44–1.57)
Ear, Nose and Throat	18	0.62	0.45	1.37 (1.23–1.53)
Urology	17	1.29	0.94	1.36 (1.26–1.47)
Gynaecology	20	0.37	0.29	1.27 (1.10–1.46)
Plastic Surgery	19	0.31	0.33	0.92 (0.79–1.07)

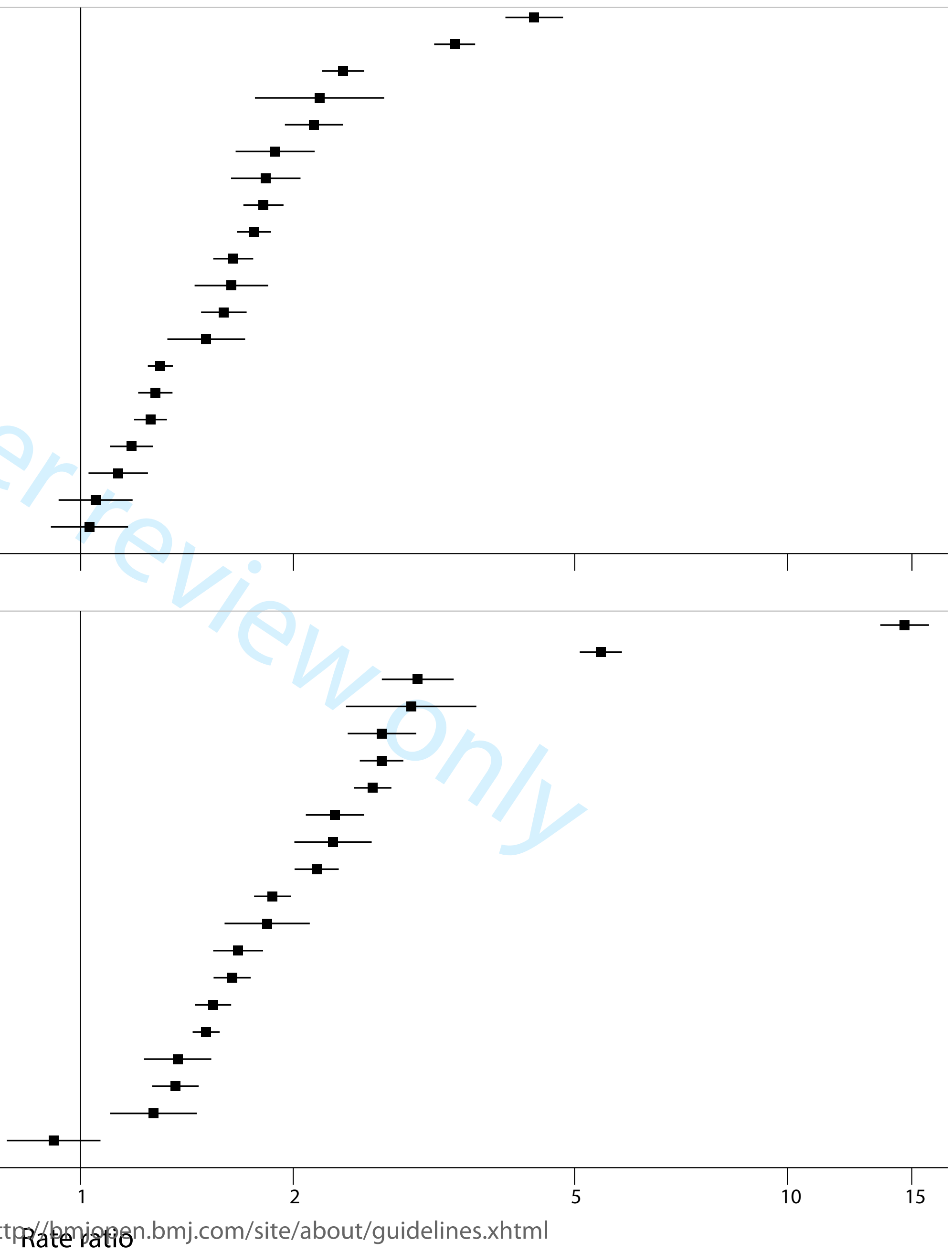

For peer review only

STROBE 2007 (v4) Statement—Checklist of items that should be included in reports of *cohort studies*

Section/Topic	Item #	Recommendation	Reported on page #
**Title and abstract**	1	(*a*) Indicate the study's design with a commonly used term in the title or the abstract	2
		(*b*) Provide in the abstract an informative and balanced summary of what was done and what was found	2
**Introduction**			
Background/rationale	2	Explain the scientific background and rationale for the investigation being reported	4
Objectives	3	State specific objectives, including any prespecified hypotheses	4
**Methods**			
Study design	4	Present key elements of study design early in the paper	5-6
Setting	5	Describe the setting, locations, and relevant dates, including periods of recruitment, exposure, follow-up, and data collection	5-6
Participants	6	(*a*) Give the eligibility criteria, and the sources and methods of selection of participants. Describe methods of follow-up	5-6
		(*b*) For matched studies, give matching criteria and number of exposed and unexposed	6
Variables	7	Clearly define all outcomes, exposures, predictors, potential confounders, and effect modifiers. Give diagnostic criteria, if applicable	7-9
Data sources/ measurement	8*	For each variable of interest, give sources of data and details of methods of assessment (measurement). Describe comparability of assessment methods if there is more than one group	5-6
Bias	9	Describe any efforts to address potential sources of bias	10-11
Study size	10	Explain how the study size was arrived at	6
Quantitative variables	11	Explain how quantitative variables were handled in the analyses. If applicable, describe which groupings were chosen and why	7-8
Statistical methods	12	(*a*) Describe all statistical methods, including those used to control for confounding	6
		(*b*) Describe any methods used to examine subgroups and interactions	
		(*c*) Explain how missing data were addressed	NA
		(*d*) If applicable, explain how loss to follow-up was addressed	NA
		(*e*) Describe any sensitivity analyses	
**Results**			

For peer review only

Participants	13*	(a) Report numbers of individuals at each stage of study—eg numbers potentially eligible, examined for eligibility, confirmed eligible, included in the study, completing follow-up, and analysed	5-6
		(b) Give reasons for non-participation at each stage	NA
		(c) Consider use of a flow diagram	NA
Descriptive data	14*	(a) Give characteristics of study participants (eg demographic, clinical, social) and information on exposures and potential confounders	8
		(b) Indicate number of participants with missing data for each variable of interest	NA
		(c) Summarise follow-up time (eg, average and total amount)	7
Outcome data	15*	Report numbers of outcome events or summary measures over time	7, Figure 1
Main results	16	(*a*) Give unadjusted estimates and, if applicable, confounder-adjusted estimates and their precision (eg, 95% confidence interval). Make clear which confounders were adjusted for and why they were included	8
		(*b*) Report category boundaries when continuous variables were categorized	8
		(*c*) If relevant, consider translating estimates of relative risk into absolute risk for a meaningful time period	
Other analyses	17	Report other analyses done—eg analyses of subgroups and interactions, and sensitivity analyses	8-9
**Discussion**			
Key results	18	Summarise key results with reference to study objectives	11-12
**Limitations**			
Interpretation	20	Give a cautious overall interpretation of results considering objectives, limitations, multiplicity of analyses, results from similar studies, and other relevant evidence	11-12
Generalisability	21	Discuss the generalisability (external validity) of the study results	11-12
**Other information**			
Funding	22	Give the source of funding and the role of the funders for the present study and, if applicable, for the original study on which the present article is based	3

*Give information separately for cases and controls in case-control studies and, if applicable, for exposed and unexposed groups in cohort and cross-sectional studies.

For peer review only

BMJ Open

Health impact of monoclonal gammopathy of undetermined significance (MGUS) and monoclonal B-cell lymphocytosis (MBL): findings from a UK population-based cohort

Journal:	*BMJ Open*
Manuscript ID	bmjopen-2020-041296.R1
Article Type:	Original research
Date Submitted by the Author:	10-Nov-2020

Complete List of Authors:	Lamb, Maxine; University of York, Department of Health Sciences<br>Smith, Alexandra; University of York, Department of Health Sciences<br>Painter, Daniel; University of York, Department of Health Sciences<br>Kane, Eleanor; University of York, Department of Health Sciences<br>Bagguley, Timothy; University of York, Department of Health Sciences<br>Newton, Robert; University of York; Uganda Virus Research Institute<br>Howell, Debra; University of York, Department of Health Sciences<br>Cook, Gordon; Leeds Teaching Hospitals NHS Trust, Haematology<br>de Tute, Ruth; Leeds Teaching Hospitals NHS Trust, HMDS<br>Rawstron, Andrew; Leeds Teaching Hospitals NHS Trust, HMDS<br>Patmore, Russell; Hull University Teaching Hospitals NHS Trust, Haematology<br>Roman, Eve; University of York, Health Sciences

**Primary Subject Heading**:	Epidemiology
Secondary Subject Heading:	Haematology (incl blood transfusion)
Keywords:	Myeloma < HAEMATOLOGY, Lymphoma < HAEMATOLOGY, EPIDEMIOLOGY, Public health < INFECTIOUS DISEASES

SCHOLARONE™
Manuscripts

I, the Submitting Author has the right to grant and does grant on behalf of all authors of the Work (as defined in the below author licence), an exclusive licence and/or a non-exclusive licence for contributions from authors who are: i) UK Crown employees; ii) where BMJ has agreed a CC-BY licence shall apply, and/or iii) in accordance with the terms applicable for US Federal Government officers or employees acting as part of their official duties; on a worldwide, perpetual, irrevocable, royalty-free basis to BMJ Publishing Group Ltd ("BMJ") its licensees and where the relevant Journal is co-owned by BMJ to the co-owners of the Journal, to publish the Work in this journal and any other BMJ products and to exploit all rights, as set out in our licence.

The Submitting Author accepts and understands that any supply made under these terms is made by BMJ to the Submitting Author unless you are acting as an employee on behalf of your employer or a postgraduate student of an affiliated institution which is paying any applicable article publishing charge ("APC") for Open Access articles. Where the Submitting Author wishes to make the Work available on an Open Access basis (and intends to pay the relevant APC), the terms of reuse of such Open Access shall be governed by a Creative Commons licence – details of these licences and which Creative Commons licence will apply to this Work are set out in our licence referred to above.

Other than as permitted in any relevant BMJ Author's Self Archiving Policies, I confirm this Work has not been accepted for publication elsewhere, is not being considered for publication elsewhere and does not duplicate material already published. I confirm all authors consent to publication of this Work and authorise the granting of this licence.

For peer review only

Health impact of monoclonal gammopathy of undetermined significance (MGUS) and monoclonal B-cell lymphocytosis (MBL): findings from a UK population-based cohort

Corresponding author: Eve Roman, Epidemiology & Cancer Statistics Group, Department of Health Sciences, Seebohm Rowntree Building, University of York, YO10 5DX, UK: eve.roman@york.ac.uk

Maxine Lamb[1], Alexandra G Smith[1], Daniel Painter[1], Eleanor Kane[1], Tim Bagguley[1], Robert Newton[1,2], Debra Howell[1], Gordon Cook[3], Ruth de Tute[4], Andrew Rawstron[4], Russell Patmore[5], Eve Roman[1]

[1] Department of Health Sciences, University of York, YO10 5DD, UK

[2] Ugandan Virus Research Institute, Entebbe, Uganda

[3] Department of Haematology, Leeds Cancer Centre, St James's University Hospital, LS9 7LP, UK

[4] Haematological Malignancy Diagnostic Service, Leeds Cancer Centre, St James's University Hospital, LS9 7LP, UK

[5] Queens Centre for Oncology and Haematology, Hull University Teaching Hospitals NHS Trust, HU16 5JQ, UK

For peer review only

For peer review only - http://bmjopen.bmj.com/site/about/guidelines.xhtml

ABSTRACT

Objective: To examine mortality and morbidity patterns before and after premalignancy diagnosis in individuals with monoclonal gammopathy of undetermined significance (MGUS) and monoclonal B-cell lymphocytosis (MBL); and compare their secondary healthcare activity to that of the general population.

Design: Population-based patient cohort, within which each patient is matched at diagnosis to 10 age and sex-matched individuals from the general population. Both cohorts are linked to nationwide information on deaths, cancer registrations, and Hospital Episode Statistics (HES).

Setting: The UK's Haematological Malignancy Research Network; which has a catchment population of around 4 million served by 14 hospitals and a central diagnostic laboratory.

Participants: All patients newly diagnosed 2009-15 with MGUS (n=2,193) or MBL (n=561), and their age and sex-matched comparators (n= 27,538).

Main Outcome measures: Mortality, and hospital inpatient and outpatient activity in the five years before, and three years after, diagnosis.

Results: Individuals with MGUS experienced excess morbidity in the 5-years before diagnosis, and excess mortality and morbidity in the 3-years after. Increased rate ratios (RR) were evident for nearly all clinical specialties; the largest, both before and after diagnosis, being for nephrology (before RR=4.29, 95% Confidence Interval 3.90-4.71; after RR=13.8, 95% CI 12.8-15.0) and rheumatology (before RR=3.40, 95% CI 3.18-3.63; after RR=5.44, 95% CI 5.08-5.83). Strong effects were also evident for endocrinology, neurology, dermatology and respiratory medicine. Conversely, only marginal increases in mortality and morbidity were evident for MBL.

Conclusions: MGUS and MBL are generally considered to be relatively benign, since most individuals with monoclonal immunoglobulins never develop a B-cell malignancy or any other M-protein related organ/tissue related disorder. Nonetheless, our findings offer strong support for the view that in some individuals monoclonal gammopathy has the potential to cause systemic disease resulting in wide-ranging organ/tissue damage and excess mortality.

For peer review only - http://bmjopen.bmj.com/site/about/guidelines.xhtml

ARTICLE SUMMARY

Strengths and limitations of this study

- Data are from an established population-based cohort within which all haematological malignancies and related clonal disorders are diagnosed, monitored, and coded at a single laboratory.
- Providing nationally generalizable data, all diagnoses are included, and complete follow-up is achieved via linkage to nationwide administrative datasets.
- The age and sex-matched general population cohort enables baseline activity and rate ratios to be calculated, both before and after premalignancy detection.
- Analyses are constrained by the fact that hospital episodes statistics are primarily collected for administrative and clinical purposes, and not for research.

Funding

This work was supported by Cancer Research UK, grant numbers 18362 and 29685; and Blood Cancer UK, grant number 15037

Competing interests

None

Data sharing agreement

Ethical approvals and data restrictions mean that data cannot be shared; but collaborative projects can be undertaken. The corresponding author can be contacted for more information.

For peer review only

BACKGROUND

Monoclonal gammopathy of undetermined significance (MGUS) and monoclonal B-cell lymphocytosis (MBL) are premalignant monoclonal B-cell disorders; the former progressing to myeloma at a rate of around 1% per year[1,2] and the latter to chronic lymphocytic leukaemia (CLL) at around 2% per year.[3,4] Diagnosed more frequently in men than women and people over 60 years of age,[5,6] overt symptoms of haematological malignancy are, by definition, absent in both MBL and MGUS.[7] Accordingly, although some premalignant disorders are found coincidentally during routine health checks, others are identified during diagnostic work-up investigations; MGUS during the course of tests applied to detect a range of potential conditions and illnesses,[8,9] and MBL during episodes of unexplained lymphocytosis.[4,10]

In addition to the association with haematological malignancy, individuals with MGUS or MBL sometimes experience higher than expected levels of mortality and morbidity that are independent of cancer.[4,8,11–16] Indeed, although most individuals with these disorders suffer no obvious ill effects, interest in their relationship with other co-morbidities has increased markedly in recent years; MBL largely in relation to its potential to impact on the immune response,[17] and MGUS due to the systemic organ and tissue damage that can be caused by monoclonal immunoglobins secreted by the abnormal B-cell clone.[18] Hitherto, however, most information about these associations has been derived either from case-control studies established to look at risk factors for disease development (e.g. family history of disease), additional tests applied to specific patient groups (e.g. patients with kidney disease), or cohort studies that track individuals with either MGUS/MBL forwards in time from their diagnosis.[5,13,18] However, despite the undoubted interest in the sequence of health events, as far as we are aware no systematic population-based investigations of the co-morbidity patterns that precede and succeed a diagnosis of either MGUS or MBL have been undertaken in the same cohort.

With a view to shedding light on the health events occurring before and after the diagnosis of MGUS and MBL, the present report uses data from an established UK population-based patient cohort of haematological malignancies and related disorders to examine the co-morbidity patterns of individuals with these premalignancy clonal disorders (MGUS=2203, MBL=561). To enable effect size quantification, these patterns are compared to the baseline activity of an individually age- and sex-matched (10 per patient) general population comparison cohort.

For peer review only

METHODS

Cases are from the Haematological Malignancy Research Network (HMRN; www.HMRN.org), a specialist UK registry established in 2004 to provide robust generalizable data to inform contemporary clinical practice and research across the country as a whole.[19,20] HMRN operates under a legal basis that permits data to be collected directly from health records without explicit consent. Set within a catchment population of around four million that is served by 14 hospitals and has a socioeconomic profile which is broadly representative of the UK as a whole, all haematological cancers and related conditions are diagnosed and coded by clinical specialists at a single integrated haematopathology laboratory, the Haematological Malignancy Diagnostic Service (www.HMDS.info), using standardized diagnostic criteria and the latest WHO ICD-O-3 classification.[7] Specifically, in relation to the present report, which covers diagnoses made in 2009-15, MBL was defined by a peripheral blood monoclonal B-cell count <5 x10^9/L in individuals with no other features of a B-cell lymphoproliferative disorder; and MGUS by a serum paraprotein less than 30 g/L, and in those where a bone marrow examination was considered necessary following clinical examination, a clonal bone marrow plasma cells/lymphoplasmacytic infiltration of less than 10%. Hence, within the HMRN region, as in other diagnostic settings [21,22], invasive bone marrow examinations in MGUS patients are generally only carried when laboratory or clinical features are indicative of an underlying plasma cell neoplasm, lymphoproliferative disorder, monoclonal immunoglobulin deposition disease (e.g. amyloidosis), or conditions like POEMS syndrome. [7]

To facilitate comparisons with unaffected individuals, HMRN also has a general population cohort. To create this 'control' cohort, all patients diagnosed 2009-15 with a haematological malignancy or related clonal disorder (n=18,127) were individually matched on sex and age at the point of diagnosis to 10 randomly selected individuals from the same catchment population. All controls were assigned a serial number that linked them to their matched case, and a "pseudo-diagnosis" date that corresponded to their matched case's diagnosis date. Individuals in the patient cohort and the comparison cohort are linked to the same nationwide information on deaths, cancer registrations and Hospital Episode Statistics (HES). At the point of selection and matching, all controls were resident in the HMRN region and none had a previous cancer registration for a haematological malignancy. [23,24]. Hence for the control cohort (in contrast to the patient cohort) no additional health information outside that contained within national administrative datasets was available.

For peer review only - http://bmjopen.bmj.com/site/about/guidelines.xhtml

Using similar methods to those previously described,[23–25] associations with hospital inpatient activity (HES Admitted Patient Care; HES-APC) and outpatient activity (HES Outpatient; HES-OP) in the five years prior to diagnosis/pseudo-diagnosis through to the three years after diagnosis were investigated. HES inpatient data contains ICD-10 (International Statistical Classification of Diseases 10th revision) codes derived from discharge summaries[26]; and associations with these were examined in relation to the 17 specific conditions in the Charlson Comorbidity Index.[27–29] By contrast, HES outpatient data contains details about the type of outpatient attendance; the majority being linked to consultant specialty codes (e.g. ophthalmology, rheumatology etc.), with the remainder largely comprising routine follow-up/monitoring, nurse-led clinic attendances (e.g. anticoagulant clinics) and consultations with allied health professionals (e.g. podiatry).

This report includes all patients (cases) who were newly diagnosed with either MGUS (n=2193) or MBL (n=561) between 1st January 2009 and 31st December 2015 and their matched controls (n=27,538); individuals diagnosed with a haematological cancer within six months of their MGUS/MBL diagnosis were considered ineligible. All cases and controls were followed-up for cancer registration and death until March 2017, and hospital activity (inpatient and outpatient) until March 2016. Additionally, progressions and/or transformations among cases were identified through HMDS up to March 2017. Data were summarised using standard methods. Overall survival, hospital activity and rate ratios (RRs) were calculated using time-to-event analyses. The Stata programme ‘strel’ was used to estimate relative survival (RS), using age- and sex-specific background mortality rates from national life tables.[30,31] All analyses were conducted using Stata 16.0.

Patient and public involvement (PPI)

PPI is integral to HMRN, and takes place via a dedicated Patient Partnership, overseen by a lay committee. Patients from the partnership are involved in identifying key research questions and participate in all our funding applications. Furthermore, patients and their relatives routinely take part in the dissemination of HMRN’s findings, which also occurs via our project website: www.yhhn.org

RESULTS

Characteristics of individuals with either MGUS or MBL are summarized alongside their corresponding controls in Table 1. Both MGUS and MBL were more commonly diagnosed in males (51.6% MGUS, 58.5% MBL) and individuals over the age of 70 years (median diagnostic age 73.4

For peer review only - http://bmjopen.bmj.com/site/about/guidelines.xhtml

years for MGUS and 72.1 years for MBL). At 90.2% (95% Confidence Interval 87.4-92.4%), the 5-year relative survival (RS) of patients diagnosed with MGUS was significantly lower ($p<0.01$) than that of their general population controls (RS 99.5%, 95% CI 98.7-99.8%). For MBL, the difference was far less marked; the 5-year RS being 94.3% (95% CI 86.3-97.7%) for MBL cases and 98.5% (95% CI 96.8-99.3%) for their controls. Interestingly, the observed variations in blood cancer progression were in the opposite direction to those seen for mortality: 75 (3.4%) MGUS patients were diagnosed with a haematological malignancy (48/75 were myelomas) before April 2017, compared to 140 (25.0%) MBL patients (137/140 were CLLs). No significant differences were, however, evident for non-haematological cancer registrations; although the frequencies were slightly higher for MGUS (8.4%) and MBL (10.3%) than for their corresponding controls (8.0% and 8.8% respectively).

For peer review only

For peer review only - http://bmjopen.bmj.com/site/about/guidelines.xhtml

Table 1: Characteristics of patients diagnosed with monoclonal gammopathy of undetermined significance (MGUS) or monoclonal B-cell lymphocytosis (MBL) and their corresponding controls; HMRN diagnoses 2009-2015

		MGUS		MBL	
		**Cases Number (%)**	**Controls Number (%)**	**Cases Number (%)**	**Controls Number (%)**
**Total**		2193 (100)	21928 (100)	561 (100)	5610 (100)
**Gender**	**Male**	1131 (51.6)	10620 (48.4)	328 (58.5)	3280 (58.5)
	**Female**	1062 (48.4)	11308 (51.6)	233 (41.5)	2330 (41.5)
**Age (years)**	**<60**	347 (15.8)	3470 (15.8)	85 (15.2)	850 (15.2)
	**60-70**	500 (22.8)	5000 (22.8)	153 (27.3)	1530 (27.3)
	**70-80**	782 (35.7)	7820 (35.7)	194 (34.6)	1940 (34.6)
	**≥80**	564 (25.7)	5638 (25.7)	129 (23.0)	1290 (23.0)
	**Median (IQR)**	73.4 (64.6 - 80.2)	73.4 (64.6 - 80.2)	72.1 (64.5 - 79.2)	72.1 (64.5 - 79.2)
**Overall survival[1]**	**5-year (95% CI)**	71.9 (69.5-74.1)	80.1 (79.4-80.7)	77.2 (72.5-81.2)	80.7 (79.4-81.9)
**Relative survival**	**5-year (95% CI)**	90.2 (87.4-92.4)	99.5 (98.7-99.8)	94.3 (86.3, 97.7)	98.5 (96.8-99.3)
**Progressions and Transformations[1]**	**Total**	75 (3.4)		140 (25.0)	
	**Myeloma**	48 (2.2)	-	-	-
	**CLL[2]**	1 (0.05)	-	137 (24.4)	-
	**Other**	26 (1.2)	-	3 (0.5)	-
**Non-haematological cancer registrations[1]**	**Total**	185 (8.4)	1748 (8.0)	58 (10.3)	494 (8.8)
	**Lung**	31 (1.5)	190 (0.9)	4 (0.7)	59 (1.1)
	**Prostate[3]**	20 (1.8)	178 (1.6)	3 (0.9)	56 (1.7)
	**Colorectal**	19 (0.9)	166 (0.8)	6 (1.1)	40 (0.7)
	**Breast[3]**	15 (1.4)	135 (1.3)	4 (1.7)	16 (0.7)
**Comorbidity score before MGUS/MBL diagnosis[4]**	**0**	1256 (57.3)	15893 (72.5)	419 (74.7)	4115 (73.4)
	**1**	506 (23.1)	3617 (16.5)	84 (15.0)	898 (16.0)
	**≥2**	431 (19.7)	2418 (11.0)	58 (10.3)	597 (10.6)
		p<0.001		p=0.78	

[1]Followed-up until 15/03/2017; [2]Chronic lymphocytic leukaemia; [3]Prostate for men only, breast for women only; [4]≥1 hospital admission with a diagnosis of one the 17 conditions included in the Charlson Comorbidity Index[25-27] during the 5 years to 1 month before MGUS/MBL diagnosis (patients)/pseudo-diagnosis (controls)

With respect to co-morbidity, in the years leading up to diagnosis MGUS patients were significantly more likely than their corresponding controls to have a record of at least one of the 17 co-morbidities specified in the Charlson Comorbidity Index[27–29] recorded in their discharge summaries; but no differences between MBL cases and their controls were evident (Table 1). More information about hospital activity patterns of cases with MGUS/MBL and their general population controls, is shown in Figure 1; which shows inpatient and outpatient activity (excluding

For peer review only

For peer review only - http://bmjopen.bmj.com/site/about/guidelines.xhtml

haematology) during the five-years before and the three-years after diagnosis of MGUS (Figure 1A) and MBL (Figure 1B). In the period before diagnosis, patients with MGUS (Figure 1A) had consistently higher outpatient activity rates than their controls, the disparity increasing markedly during the 18 months leading up to the formal diagnosis of MGUS by haematopathology where it remained high for about 12 months, before gradually falling and levelling out at a higher level than before diagnosis. Although less pronounced, a similar pattern is evident in inpatient data. With smaller numbers and more scatter, variations in outpatient and inpatient activity in MBL are less evident (Figure 1B).

Figure 2 shows outpatient attendance frequencies (at least two specialty-specific visits) in the three years before and in the three years after MGUS diagnosis for the top 25 clinical specialties; visits within one month (±) of diagnosis/pseudodiagnosis are excluded. As is evident from the plot, the increased outpatient activity seen among cases (Figure 1) occurs across a range of clinical specialties; the highest frequencies occurring in ophthalmology, haematology, general surgery, orthopaedics, general (internal) medicine and rheumatology. However, excluding haematology where, as expected, attendances increased markedly just before and after MGUS diagnosis, the largest rate ratios (RR) both before (Figure 3A) and after (Figures 3B) diagnosis were for nephrology (before diagnosis RR= 4.29, 95% CI 3.90-4.71; after diagnosis RR= 13.8, 95% CI 12.8-15.0) and rheumatology (before diagnosis RR= 3.40, 95% CI 3.18-3.63; after diagnosis RR= 5.44, 95% CI 5.08-5.83). Other significant associations ($p<0.05$) with rate ratio point estimates above 2.0 were evident for endocrinology, neurology, and respiratory medicine, as well as for the nurse-led monitoring activities which form part of on-going clinical care across a range of specialties.

MGUS data are stratified by subtype in Table 2. Accounting for around two-thirds of the total (n=1471; 67.0%) the IgG subtype dominates, followed by IgM (n=350; 16.0%) and IgA (n=266; 12.1%). The remaining 106 (4.8%) 'other' category comprise a mix of subtypes: light chain only (n=60), IgG + IgM (n=17), IgG + IgA (n=6), IgA + IgM (n=1), IgE (n=2); and not recorded (n=20). As expected, progression to myeloma in the three years following MGUS diagnosis was largely restricted to the IgG and IgA subtypes. The age distributions, five-year survival estimates (overall and relative) and non-haematological malignancy frequencies of the main subtypes were broadly similar; although patients in the combined 'other' category tended to be slightly older and to fare less well (RS= 77.6%, 95% CI 62.6-87.2%). The numbers of patients in the individual groups were, however, too sparse to examine the data in greater depth. Finally, during the study period (2009-15), in addition to the detection of a paraprotein in peripheral blood, around 70% (1527/2193) of MGUS

For peer review only - http://bmjopen.bmj.com/site/about/guidelines.xhtml

For peer review only

patients in our cohort had a confirmatory bone marrow examination taken to exclude an underlying neoplasm. The patient characteristics and secondary care activity patterns of those who had bone marrow examinations were, however, broadly similar to those who did not (data not shown).

For peer review only

For peer review only - http://bmjopen.bmj.com/site/about/guidelines.xhtml

Table 2: Characteristics of patients diagnosed with monoclonal gammopathy of undetermined significance (MGUS) and their corresponding controls, stratified by MGUS subtype; HMRN diagnoses 2009-2015

	MGUS subtype							
	IgG		IgM		IgA		Other[1]	
	Cases Number (%)	Controls Number (%)	Cases Number (%)	Controls Number (%)	Cases Number (%)	Controls Number (%)	Cases Number (%)	Controls Number (%)
**Total**	1471 (100.0)	14709 (100.0)	350 (100.0)	3499 (100.0	266 (100.0)	2660 (100.0)	106 (100.0)	1060 (100.0)
**Gender**								
**Male**	745 (50.6)	7449 (50.6)	177 (50.6)	1769 (50.6)	145 (54.5)	1450 (54.5)	64 (60.4)	640 (60.4)
**Female**	726 (49.4)	7260 (49.4)	173 (49.4)	1730 (49.4)	121 (45.5)	1210 (45.5)	42 (39.6)	420 (39.6)
**Age (years)**								
**<60**	239 (16.2)	2390 (16.2)	50 (14.3)	500 (14.3)	48 (18.0)	480 (18.0)	10 (9.4)	100 (9.4)
**60-70**	336 (22.8)	3360 (22.8)	80 (22.9)	800 (22.9)	56 (21.1)	560 (21.1)	28 (26.4)	280 (26.4)
**70-80**	514 (34.9)	5140 (34.9)	130 (37.1)	1300 (37.2)	104 (39.1)	1040 (39.1)	34 (32.1)	340 (32.1)
**≥80**	382 (26.0)	3819 (26.0)	90 (25.7)	899 (25.7)	58 (21.8)	580 (21.8)	34 (32.1)	340 (32.1)
**Median**	73.4	73.4	73.0	73.0	73.0	73.0	75.3	75.3
**Survival 5-year (95% CI)[2]**								
**Overall**	72.3 (69.4-74.9)	79.5 (78.7-80.3)	69.6 (63.3-75.0)	81.3 (79.7-82.9)	74.2 (67.3-79.8)	82.4 (80.6-84.1)	68.6 (56.5-78.0)	77.6 (74.1-80.7)
**Relative**	90.6 (86.9-93.2)	99.2 (98.3-99.7)	84.2 (75.8-89.8)	99.3 (93.7-99.9)	88.3 (78.5-93.8)	98.5 (95.3-99.5)	77.6 (62.6-87.2)	96.8 (89.5-99.1)
**Progressions/transformations[2]**								
**Total**	44 (3.0)	-	8 (2.3)	-	17 (6.4)	-	6 (5.7)	-
**Myeloma**	34 (2.3)	-	1 (0.3)	-	11 (4.1)	-	2 (1.9)	-
**Other**	10 (0.7)	-	7 (2.0)	-	6 (2.3)	-	4 (3.8)	-
**Non-haematological cancer registrations[2]**								
**Total**	118 (8.0)	1173(8.0)	41 (11.7)	277 (7.9)	19 (7.1)	210 (7.9)	7 (6.6)	88 (8.4)
**Lung**	16 (1.1)	133 (0.9)	6 (1.7)	24 (0.7)	4 (1.5)	21 (0.8)	5 (4.7)	12 (1.1)
**Prostate[3]**	14 (1.9)	115 (1.5)	3 (1.7)	27 (1.5)	3 (2.1)	25 (1.7)	-	11 (1.7)
**Colorectal**	16 (1.1)	112 (0.8)	2 (0.6)	26 (0.7)	1 (0.4)	20 (0.7)	-	8 (0.8)
**Breast[3]**	9 (1.2)	88 (1.2)	4 (2.3)	26 (1.5)	2 (1.7)	15 (1.2)	-	6 (1.4)
**Hospital outpatient activity (≥ 3 visits), excluding haematology and visits within 1 month of diagnosis[4]**								
**5-years before diagnosis**	1173 (79.7)	9103 (61.9)	287 (82.0)	2155 (61.6)	216 (81.2)	1633 (61.4)	89 (84.0)	663 (62.5)
**3-years after diagnosis**	964 (65.5)	6558 (44.6)	240 (68.6)	1577 (45.1)	176 (66.2)	1154 (43.4)	70 (66.0)	445 (42.0)

[1]Biclonal, IgD, IgE, light chain, not known; [2]Followed-up until 15/03/2017; [3]Prostate for men only, breast for women only; [4]In the 5 years before diagnosis/pseudo-diagnosis and 3 years after diagnosis/pseudo-diagnosis

For peer review only

For peer review only - http://bmjopen.bmj.com/site/about/guidelines.xhtml

DISCUSSION

Including data on nearly 3000 cases with pre-malignant clonal disorders and ten times as many age and sex-matched general population controls, this large UK record-linkage study found that individuals with MGUS not only experienced excess mortality and morbidity after diagnosis, but also excess morbidity in the 5 years before. By contrast, only marginal increases in mortality and morbidity were evident for MBL; none of which were consistent or varied significantly from the general population. Interestingly, progression patterns were in the opposite direction: in the years following detection of a pre-malignant clonal disorder, 3.4% (n=75/2193) of those with MGUS developed a haematological malignancy (41 of which were myelomas) before April 2017, compared to 25.0% (n=140/561) of those with MBL (137 of which were CLLs).

The elevated mortality and morbidity following a diagnosis of MGUS, which seems largely independent of progression to cancer, is consistent with reports relating to the potential clinical significance of this disorder.[11–18] Corresponding to the period of diagnostic work-up, our data also demonstrate the pronounced increase in hospital activity in the months surrounding MGUS diagnosis; the highest activity being observed in the six months before and six months after diagnosis. Of more importance, perhaps, the analyses clearly show that hospital activity in people subsequently diagnosed with MGUS is often elevated many years before diagnosis: excesses being observed in specialties covering most organ and tissue systems including nephrology, endocrinology, neurology, rheumatology, gastroenterology, dermatology and respiratory medicine. By contrast, although hospital activity increased in the months around the time of MBL diagnosis, no consistent differences or patterns either before or after then, were detected. Furthermore, in agreement with findings reported in other studies that used age and sex-matched controls, no associations with mortality were detected.[3,32] However, given the fact that MBL has been associated with increased susceptibility to infection and non-CLL related mortality [4,15,33], it is possible that the findings relating to subsequent morbidity and mortality could change as our data mature, length of follow-up increases, and linkage to primary care data becomes possible.

The age and sex distributions of our population-based cohorts are broadly similar to those of other published MBL [3,34,35] and MGUS [1,2,11] series; as is the dominance of the IgG MGUS subtype.[1,2] Providing nationally generalizable data, additional strengths of our study include its large well-defined population, within which all haematological malignancies and related clonal disorders are diagnosed, monitored, and coded using up-to-date standardized procedures at a central haematopathology laboratory.[19] In this context, it is important to bear in mind that most people with premalignant clonal disorders remain asymptomatic, and that our cohorts contain a relatively large proportion of people who came to clinical attention in primary and/or secondary care and were referred to haematology for further investigation. More specifically, around 98% of patients in our MBL cohort had high-count MBL, and within the HMRN region MBL patients are monitored routinely using flow cytometry so laboratory progressions

For peer review only

(B-cell count >5 x10^{9}/L) may be detected with higher sensitivity than in cases monitored clinically. This is supported by the fact that over the follow-up period (median 3.8 years), around a quarter of MBL patients progressed to CLL, and 3.6% required treatment. Prevalence comparisons with population screening studies also confirm that the majority of those over 50 years of age with monoclonal immunoglobulin in their blood/urine would not be included in our MGUS cohort.[5,20,35–37] Furthermore, in the context of population screening, it is also important to remember that some members of the control cohort would, if screened, be found to have premalignant clonal disorders detected in their peripheral blood . [13,32,33,38,39].

The diversity of morbidity effects seen among individuals with MGUS is consistent with the expanding body of evidence relating to the potential adverse impact that even low levels of circulating monoclonal protein (M-protein) can have. Thus far, the complex underpinning mechanisms identified include; deposition of M-protein aggregations of varying immunoglobulin subtypes in different organs, as well as the induction of auto-antibodies and cytokines that can impact on organs and tissues in a variety of deleterious ways.[13,18,40,41] Indeed, the recognized number of M-protein mediated entities is increasing, with several affecting multiple organs; well-known deposition syndromes including primary amyloidosis and paraneoplastic conditions such as POEMS syndrome (polyneuropathy, organomegaly, endocrinopathy, monoclonal gammopathy, and skin changes).[7,42] As evidenced in our analysis, kidney involvement is frequent, both in the years before (four-fold excess) and after (fourteen-fold excess) MGUS diagnosis. Indeed, the umbrella term monoclonal gammopathy of renal significance (MGRS) has recently been suggested to cover all M-protein mediated kidney disorders that fail to meet the diagnostic criteria for multiple myeloma or any other B-cell malignancy.[13,18,43] Other organ-specific terms continue to emerge, and with a view to improving recognition of these complex disorders which clearly pose significant diagnostic and treatment challenges, the overarching term monoclonal gammopathy of clinical significance (MGCS) has also been suggested.[18]

From a haematological malignancy perspective, MGUS and MBL are generally considered to be relatively benign conditions. However, both can have other deleterious health consequences; the effect of monoclonal gammopathy being particularly striking. Impacting significantly on survival and having the potential to cause systemic disease and wide-ranging damage to most organs and tissues, the adverse outcomes associated with the M-proteins produced by the abnormal B-cell clone can be severe and extend over many years. Even though most people with monoclonal immunoglobulins never develop a B-cell malignancy or suffer from any other form of M-protein related organ/tissue related disorder, the consequences for those that do can be extremely serious. In this regard, early targeting of pathogenic B-cell clones could mitigate both cancer and non-cancer effects; but currently, although knowledge is increasing, there is no known way to reliably identify such clones in the absence of other signs/symptoms.

For peer review only

Hence, population screening cannot be recommended, and diagnosis remains reliant on clinical suspicion. However, the long-standing nature of the co-morbidity associations seen prior to MGUS diagnosis in our data, suggest that there may be room for improvement, and that the implementation of strategies to improve awareness and earlier detection, as well as monitoring of high-risk patient groups, could prove beneficial.

Ethics Approval: The Haematological Malignancy Research Network has ethics approval (REC 04/01205/69) from Leeds West Ethics Committee, R&D approval from each NHS Trust, and exemption from Section 251 of the Health & Social Care Act (PIAG 1-05 9h)/2007)

Contributors: ER, AS, DH and RP initiated the patient cohort within which this research is nested; and ER, AS, and EK designed the comparison cohort. ER, AS and ML planned this study and analyses. R de T and AR oversaw all laboratory procedures; and ML, DP, EK, TB managed the data and carried out data analysis. GC, RP, AR, and RN commented on the biological and clinical aspects. ER and ML drafted the manuscript, which was approved by all authors.

References

1 Kyle RA, Larson DR, Therneau TM, *et al.* Long-Term Follow-up of Monoclonal Gammopathy of Undetermined Significance. *N Engl J Med* 2018;**378**:241–9. doi:10.1056/NEJMoa1709974

2 Turesson I, Kovalchik SA, Pfeiffer RM, *et al.* Monoclonal gammopathy of undetermined significance and risk of lymphoid and myeloid malignancies: 728 cases followed up to 30 years in Sweden. *Blood* 2014;**123**:338–45. doi:10.1182/blood-2013-05-505487

3 Parikh SA, Chaffee KG, Larson MC, *et al.* Outcomes of a large cohort of individuals with clinically ascertained high-count monoclonal B-cell lymphocytosis. *Haematologica* 2018;**103**:e237–40. doi:10.3324/haematol.2017.183194

4 Rawstron AC, Bennett FL, O'Connor SJM, *et al.* Monoclonal B-cell lymphocytosis and chronic lymphocytic leukemia. *N Engl J Med* 2008;**359**:575–83. doi:10.1056/NEJMoa075290

5 Goldin LR, McMaster ML, Caporaso NE. Precursors to lymphoproliferative malignancies. *Cancer Epidemiol Biomarkers Prev* 2013;**22**:533–9. doi:10.1158/1055-9965.EPI-12-1348

6 Smith A, Howell D, Patmore R, *et al.* Incidence of haematological malignancy by sub-type: a report from the Haematological Malignancy Research Network. *Br J Cancer* 2011;**105**:1684–92. doi:10.1038/bjc.2011.450

7 Swerdlow S, Campo E, Harris N, *et al. WHO Classification of Tumours of Haematopoietic and Lymphoid Tissues*. Lyon, France: : World Health Organization 2017.

8 Kristinsson SY, Björkholm M, Andersson TM-L, *et al.* Patterns of survival and causes of death following a diagnosis of monoclonal gammopathy of undetermined significance: a population-based study. *Haematologica* 2009;**94**:1714–20. doi:10.3324/haematol.2009.010066

For peer review only

9 Merlini G, Palladini G. Differential diagnosis of monoclonal gammopathy of undetermined significance. *Hematology Am Soc Hematol Educ Program* 2012;**2012**:595–603. doi:10.1182/asheducation.V2012.1.595.3798563

10 Shanafelt TD, Ghia P, Lanasa MC, *et al.* Monoclonal B-cell lymphocytosis (MBL): biology, natural history and clinical management. *Leukemia* 2010;**24**:512–20. doi:10.1038/leu.2009.287

11 Kristinsson SY, Tang M, Pfeiffer RM, *et al.* Monoclonal gammopathy of undetermined significance and risk of infections: a population-based study. *Haematologica* 2012;**97**:854–8.

12 Thorsteinsdottir S, Lund SH, Lindqvist EK, *et al.* Bone disease in monoclonal gammopathy of undetermined significance: results from a screened population-based study. *Blood Adv* 2017;**1**:2790–8. doi:10.1182/bloodadvances.2017010454

13 Glavey SV, Leung N. Monoclonal gammopathy: The good, the bad and the ugly. *Blood Rev* 2016;**30**:223–31. doi:10.1016/j.blre.2015.12.001

14 Ciocchini M, Arbelbide J, Musso CG. Monoclonal gammopathy of renal significance (MGRS): the characteristics and significance of a new meta-entity. *Int Urol Nephrol* 2017;**49**:2171–5. doi:10.1007/s11255-017-1594-y

15 Moreira J, Rabe KG, Cerhan JR, *et al.* Infectious complications among individuals with clinical monoclonal B-cell lymphocytosis (MBL): a cohort study of newly diagnosed cases compared to controls. *Leukemia* 2013;**27**:136–41.

16 Rögnvaldsson S, Steingrímsson V, Turesson I, *et al.* Peripheral neuropathy and monoclonal gammopathy of undetermined significance: a population-based study including 15,351 cases and 58,619 matched controls. *Haematologica* Published Online First: 13 February 2020. doi:10.3324/haematol.2019.239632

17 Criado I, Blanco E, Rodríguez-Caballero A, *et al.* Residual normal B-cell profiles in monoclonal B-cell lymphocytosis versus chronic lymphocytic leukemia. *Leukemia* 2018;**32**:2701–5. doi:10.1038/s41375-018-0164-3

18 Fermand J-P, Bridoux F, Dispenzieri A, *et al.* Monoclonal gammopathy of clinical significance: a novel concept with therapeutic implications. *Blood* 2018;**132**:1478–85. doi:10.1182/blood-2018-04-839480

19 Smith A, Howell D, Crouch S, *et al.* Cohort Profile: The Haematological Malignancy Research Network (HMRN); a UK population-based patient cohort. *Int J Epidemiol* Published Online First: 2018. doi:10.1093/ije/dyy044

20 Haematological Malignancy Research Network. HMRN - Statistics. https://www.hmrn.org/statistics (accessed 12 Apr 2020).

21 Kyle RA, Therneau TM, Rajkumar SV, *et al.* A long-term study of prognosis in monoclonal gammopathy of undetermined significance. *N Engl J Med* 2002;**346**:564–9. doi:10.1056/NEJMoa01133202

22 Landgren O, Kristinsson SY, Goldin LR, *et al.* Risk of plasma cell and lymphoproliferative disorders among 14621 first-degree relatives of 4458 patients with monoclonal gammopathy of undetermined significance in Sweden. *Blood* 2009;**114**:791–5. doi:10.1182/blood-2008-12-191676

23 Kane E, Painter D, Smith A, *et al.* The impact of rheumatological disorders on lymphomas and myeloma: a report on risk and survival from the UK's population-based Haematological Malignancy Research Network. *Cancer Epidemiology* 2019;**59**:236–43. doi:10.1016/j.canep.2019.02.014

For peer review only

24 Kane E, Painter D, Smith A, *et al.* Risk of mature B-cell neoplasms and precursor conditions after joint replacement: a report from the Haematological Malignancy Research Network. *Int J Cancer* Published Online First: 1 November 2019. doi:10.1002/ijc.32765

25 Lamb MJ, Roman E, Howell DA, *et al.* Hodgkin lymphoma detection and survival: findings from the Haematological Malignancy Research Network. *BJGP Open* 2019;**3**. doi:10.3399/bjgpopen19X101668

26 World Health Organization. International Statistical Classification of Diseases and Related Health Problems, 10th revision. 2010.

27 Maringe C, Fowler H, Rachet B, *et al.* Reproducibility, reliability and validity of population-based administrative health data for the assessment of cancer non-related comorbidities. *PLOS ONE* 2017;**12**:e0172814. doi:10.1371/journal.pone.0172814

28 Wästerlid T, Mohammadi M, Smedby KE, *et al.* Impact of comorbidity on disease characteristics, treatment intent and outcome in diffuse large B-cell lymphoma: a Swedish lymphoma register study. *Journal of Internal Medicine* 2019;**285**:455–68. doi:10.1111/joim.12849

29 Charlson ME, Pompei P, Ales KL, *et al.* A new method of classifying prognostic comorbidity in longitudinal studies: Development and validation. *Journal of Chronic Diseases* 1987;**40**:373–83. doi:10.1016/0021-9681(87)90171-8

30 Estève J, Benhamou E, Croasdale M, *et al.* Relative survival and the estimation of net survival: Elements for further discussion. *Statist Med* 1990;**9**:529–38. doi:10.1002/sim.4780090506

31 Cancer Research UK Cancer Survival Group. UK life tables. UK life tables. 2017.http://csg.lshtm.ac.uk/tools-analysis/uk-life-tables/ (accessed 26 Jan 2018).

32 Strati P, Shanafelt TD. Monoclonal B-cell lymphocytosis and early-stage chronic lymphocytic leukemia: diagnosis, natural history, and risk stratification. *Blood* 2015;**126**:454–62. doi:10.1182/blood-2015-02-585059

33 Criado I, Rodríguez-Caballero A, Gutiérrez ML, *et al.* Low-count monoclonal B-cell lymphocytosis persists after seven years of follow up and is associated with a poorer outcome. *Haematologica* 2018;**103**:1198–208. doi:10.3324/haematol.2017.183954

34 Rawstron AC, Shanafelt T, Lanasa MC, *et al.* Different biology and clinical outcome according to the absolute numbers of clonal B-cells in monoclonal B-cell lymphocytosis (MBL). *Cytometry B Clin Cytom* 2010;**78 Suppl 1**:S19-23. doi:10.1002/cyto.b.20533

35 Call TG, Norman AD, Hanson CA, *et al.* Incidence of chronic lymphocytic leukemia and high-count monoclonal B-cell lymphocytosis using the 2008 guidelines. *Cancer* 2014;**120**:2000–5. doi:10.1002/cncr.28690

36 Wadhera RK, Rajkumar SV. Prevalence of monoclonal gammopathy of undetermined significance: a systematic review. *Mayo Clin Proc* 2010;**85**:933–42. doi:10.4065/mcp.2010.0337

37 Li J, Smith A, Crouch S, *et al.* Estimating the prevalence of hematological malignancies and precursor conditions using data from Haematological Malignancy Research Network (HMRN). *Cancer Causes Control* 2016;**27**:1019–26. doi:10.1007/s10552-016-0780-z

38 Kyle RA, Therneau TM, Rajkumar SV, *et al.* Prevalence of monoclonal gammopathy of undetermined significance. *N Engl J Med* 2006;**354**:1362–9. doi:10.1056/NEJMoa054494

For peer review only

39 Landgren O, Graubard BI, Kumar S, *et al.* Prevalence of myeloma precursor state monoclonal gammopathy of undetermined significance in 12372 individuals 10-49 years old: a population-based study from the National Health and Nutrition Examination Survey. *Blood Cancer J* 2017;**7**:e618. doi:10.1038/bcj.2017.97

40 Merlini G, Stone MJ. Dangerous small B-cell clones. *Blood* 2006;**108**:2520–30. doi:10.1182/blood-2006-03-001164

41 Atkin C, Richter A, Sapey E. What is the significance of monoclonal gammopathy of undetermined significance? *Clin Med (Lond)* 2018;**18**:391–6. doi:10.7861/clinmedicine.18-5-391

42 Dispenzieri A. POEMS Syndrome: 2019 Update on diagnosis, risk-stratification, and management. *Am J Hematol* 2019;**94**:812–27. doi:10.1002/ajh.25495

43 Amaador K, Peeters H, Minnema MC, *et al.* Monoclonal gammopathy of renal significance (MGRS) histopathologic classification, diagnostic workup, and therapeutic options. *Neth J Med* 2019;**77**:243–54.

Figure Legends

Figure 1: Inpatient (IP) and outpatient (OP) monthly hospital visit activity rates five years before to three years after diagnosis (cases)/pseudo-diagnosis (controls) excluding haematology attendances: A) monoclonal gammopathy of undetermined significance (MGUS) and B) monoclonal B-cell lymphocytosis (MBL) diagnosed 2009-15

Figure 2: Percentage of cases and controls with at least two specialty-specific outpatient visits in the three years before and after diagnosis of monoclonal gammopathy of undetermined significance (MGUS). Top 25 recorded specialties, with visits within one month of diagnosis/pseudodiagnosis excluded

Figure 3: Monthly outpatient attendance rates (per 100 persons) in cases and controls and rate ratios by outpatient specialty with at least two visits (A) in the three years before diagnosis and (B) in the three years after diagnosis of monoclonal gammopathy of undetermined significance (MGUS)

For peer review only

Figure 1: Inpatient (IP) and outpatient (OP) monthly hospital visit activity rates five years before to three years after diagnosis (cases)/pseudo-diagnosis (controls) excluding haematology attendances: A) monoclonal gammopathy of undetermined significance (MGUS) and B) monoclonal B-cell lymphocytosis (MBL) diagnosed 2009-15

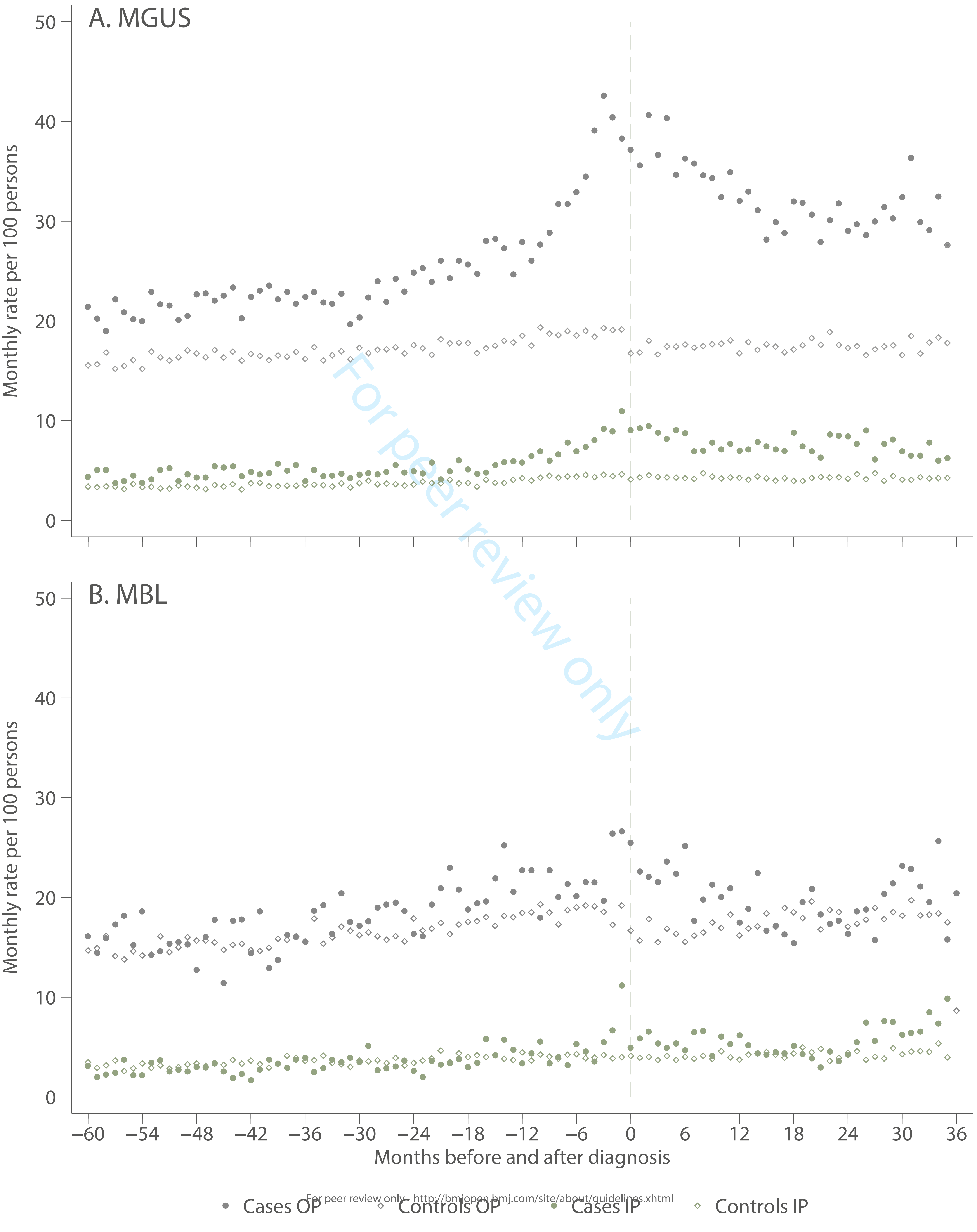

For peer review only

Figure 2: Percentage of cases and controls with at least two specialty-specific outpatient visits in the three years before and after diagnosis of monoclonal gammopathy of undetermined significance (MGUS). Top 25 recorded specialties, with visits within one month of diagnosis/pseudodiagnosis excluded.

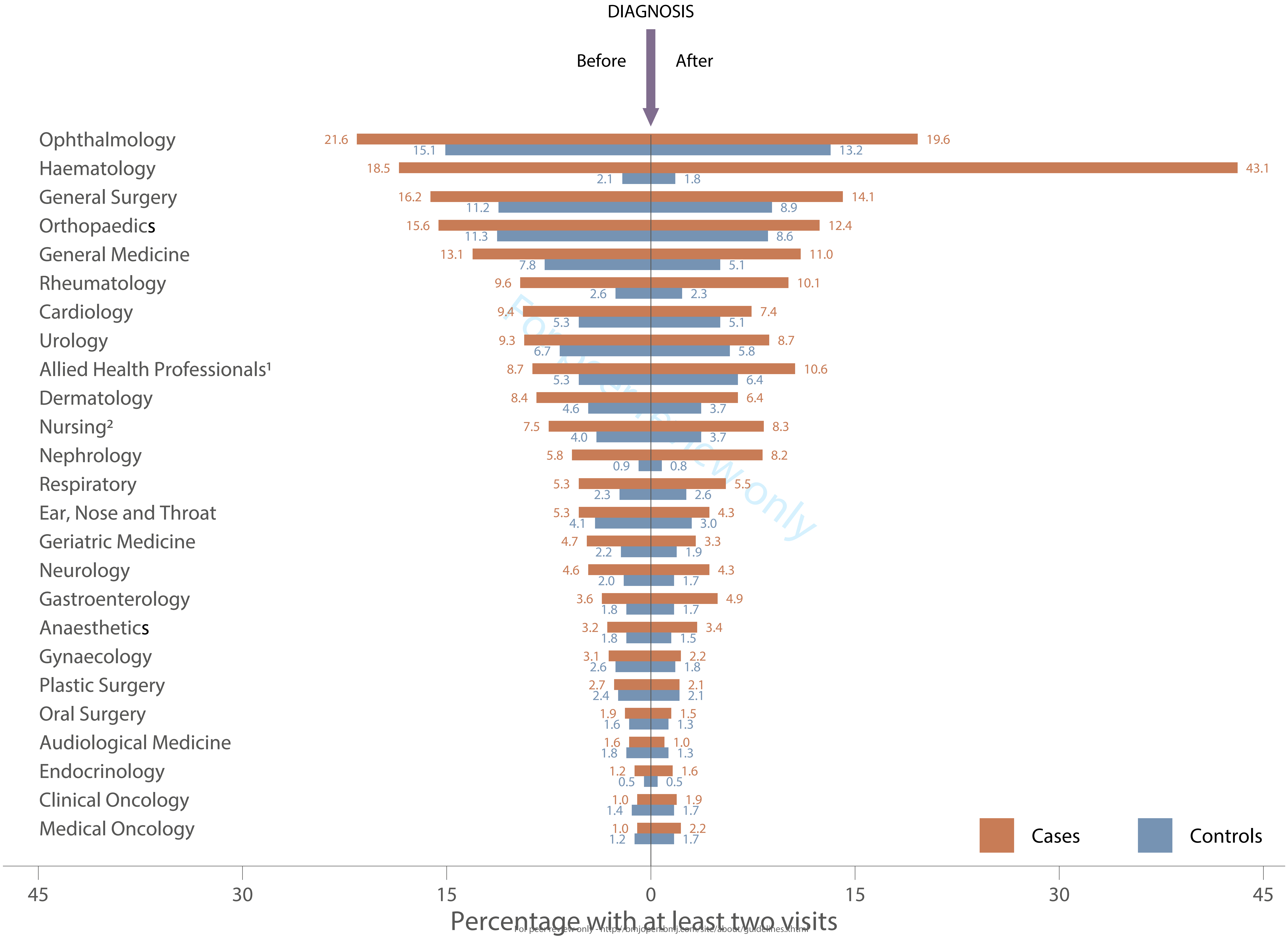

Figure 3: Monthly outpatient attendance rates (per 100 persons) in cases and controls and rate ratios by outpatient specialty with at least two visits (A) in the three years before diagnosis and (B) in the three years after diagnosis of monoclonal gammopathy of undetermined significance (MGUS)

Outpatient specialty	Post–diagnosis rank	Case rate	Control rate	Rate ratio (95% CI)
Nephrology	1	0.91	0.21	4.29 (3.90–4.71)
Rheumatology	2	1.70	0.50	3.40 (3.18–3.63)
Nursing	6	1.45	0.62	2.34 (2.19–2.51)
Endocrinology	4	0.15	0.07	2.19 (1.77–2.70)
Respiratory	8	0.74	0.35	2.11 (1.92–2.32)
Gastroenterology	3	0.40	0.21	1.90 (1.67–2.16)
Neurology	5	0.52	0.28	1.83 (1.63–2.05)
Dermatology	10	1.54	0.85	1.81 (1.70–1.93)
General Medicine	7	2.57	1.48	1.77 (1.67–1.87)
Allied Health Professionals	11	1.53	0.93	1.65 (1.55–1.76)
Geriatric Medicine	12	0.45	0.28	1.63 (1.44–1.83)
Cardiology	13	1.16	0.74	1.57 (1.46–1.69)
Anaesthetics	9	0.40	0.26	1.51 (1.33–1.71)
Ophthalmology	16	3.78	2.92	1.30 (1.25–1.35)
General Surgery	14	1.99	1.57	1.27 (1.20–1.34)
Orthopaedics	15	2.15	1.74	1.24 (1.17–1.31)
Urology	18	1.27	1.08	1.18 (1.10–1.26)
Ear, Nose and Throat	17	0.66	0.58	1.13 (1.02–1.24)
Plastic Surgery	20	0.42	0.40	1.07 (0.95–1.21)
Gynaecology	19	0.38	0.37	1.03 (0.90–1.16)

Outpatient specialty	Pre–diagnosis rank	Case rate	Control rate	Rate ratio (95% CI)
Nephrology	1	2.41	0.17	13.8 (12.8–15.0)
Rheumatology	2	2.07	0.39	5.44 (5.08–5.83)
Gastroenterology	6	0.61	0.20	3.03 (2.69–3.40)
Endocrinology	4	0.19	0.06	2.95 (2.39–3.65)
Neurology	7	0.66	0.25	2.64 (2.36–2.95)
Nursing	3	1.30	0.59	2.63 (2.45–2.82)
General Medicine	9	2.54	1.06	2.56 (2.41–2.72)
Respiratory	5	0.88	0.39	2.28 (2.07–2.50)
Anaesthetics	13	0.51	0.22	2.33 (2.06–2.65)
Dermatology	8	1.53	0.71	2.16 (2.01–2.32)
Allied Health Professionals	10	2.13	1.14	1.87 (1.76–1.98)
Geriatric Medicine	11	0.40	0.22	1.84 (1.60–2.11)
Cardiology	12	1.15	0.69	1.66 (1.53–1.80)
General Surgery	15	2.08	1.27	1.64 (1.54–1.74)
Orthopaedics	16	2.15	1.39	1.54 (1.45–1.64)
Ophthalmology	14	3.90	2.58	1.51 (1.44–1.58)
Ear, Nose and Throat	18	0.62	0.45	1.38 (1.24–1.54)
Urology	17	1.28	0.94	1.36 (1.26–1.47)
Gynaecology	20	0.37	0.29	1.27 (1.10–1.46)
Plastic Surgery	19	0.31	0.33	0.92 (0.79–1.07)

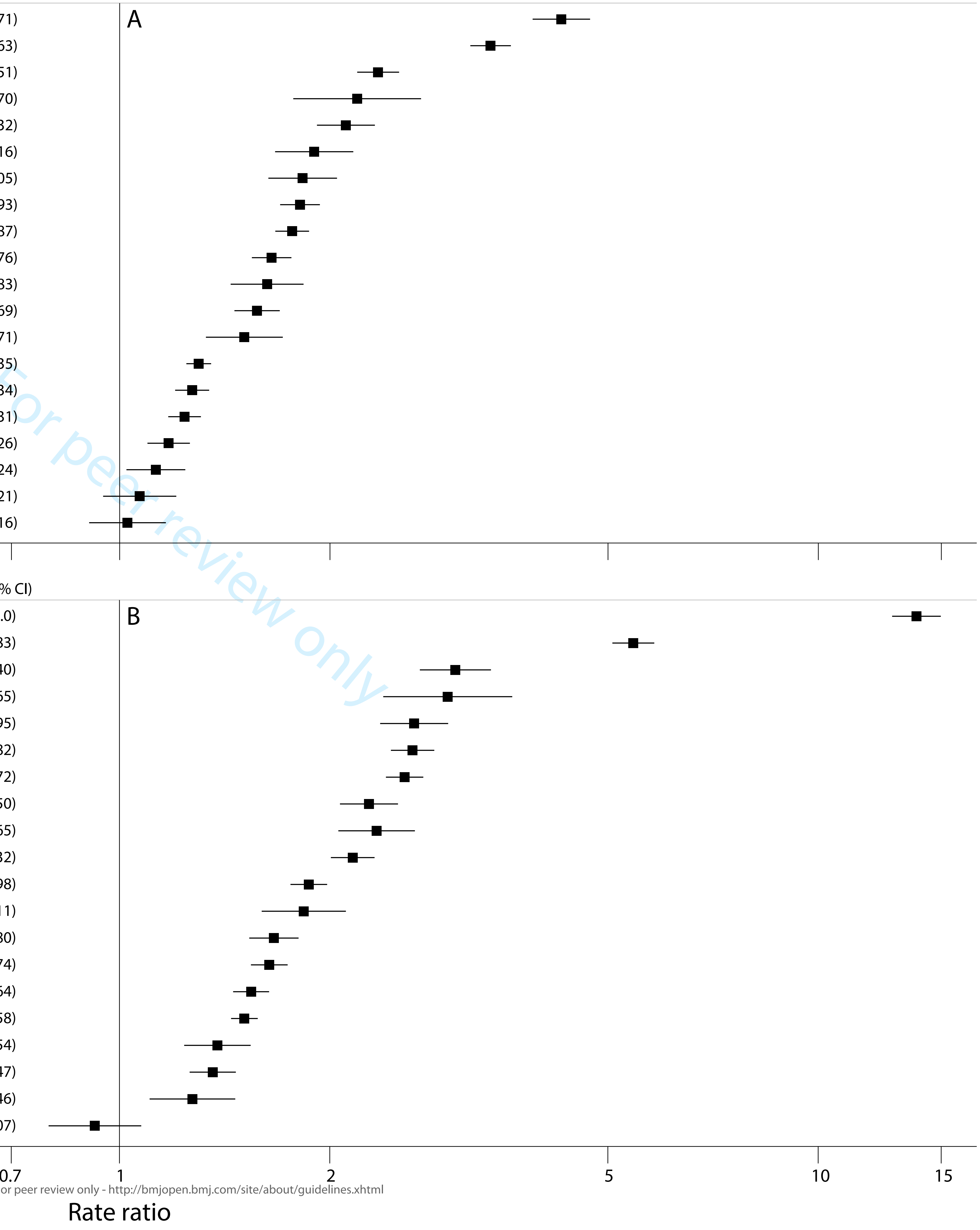

For peer review only

BMJ Open

Health impact of monoclonal gammopathy of undetermined significance (MGUS) and monoclonal B-cell lymphocytosis (MBL): findings from a UK population-based cohort

Journal:	*BMJ Open*
Manuscript ID	bmjopen-2020-041296.R2
Article Type:	Original research
Date Submitted by the Author:	20-Jan-2021
Complete List of Authors:	Lamb, Maxine; University of York, Department of Health Sciences<br>Smith, Alexandra; University of York, Department of Health Sciences<br>Painter, Daniel; University of York, Department of Health Sciences<br>Kane, Eleanor; University of York, Department of Health Sciences<br>Bagguley, Timothy; University of York, Department of Health Sciences<br>Newton, Robert; University of York; Uganda Virus Research Institute<br>Howell, Debra; University of York, Department of Health Sciences<br>Cook, Gordon; Leeds Teaching Hospitals NHS Trust, Haematology<br>de Tute, Ruth; Leeds Teaching Hospitals NHS Trust, HMDS<br>Rawstron, Andrew; Leeds Teaching Hospitals NHS Trust, HMDS<br>Patmore, Russell; Hull University Teaching Hospitals NHS Trust, Haematology<br>Roman, Eve; University of York, Health Sciences
**Primary Subject Heading**:	Epidemiology
Secondary Subject Heading:	Haematology (incl blood transfusion)
Keywords:	Myeloma < HAEMATOLOGY, Lymphoma < HAEMATOLOGY, EPIDEMIOLOGY, Leukaemia < HAEMATOLOGY, PUBLIC HEALTH

SCHOLARONE™
Manuscripts

I, the Submitting Author has the right to grant and does grant on behalf of all authors of the Work (as defined in the below author licence), an exclusive licence and/or a non-exclusive licence for contributions from authors who are: i) UK Crown employees; ii) where BMJ has agreed a CC-BY licence shall apply, and/or iii) in accordance with the terms applicable for US Federal Government officers or employees acting as part of their official duties; on a worldwide, perpetual, irrevocable, royalty-free basis to BMJ Publishing Group Ltd ("BMJ") its licensees and where the relevant Journal is co-owned by BMJ to the co-owners of the Journal, to publish the Work in this journal and any other BMJ products and to exploit all rights, as set out in our licence.

The Submitting Author accepts and understands that any supply made under these terms is made by BMJ to the Submitting Author unless you are acting as an employee on behalf of your employer or a postgraduate student of an affiliated institution which is paying any applicable article publishing charge ("APC") for Open Access articles. Where the Submitting Author wishes to make the Work available on an Open Access basis (and intends to pay the relevant APC), the terms of reuse of such Open Access shall be governed by a Creative Commons licence – details of these licences and which Creative Commons licence will apply to this Work are set out in our licence referred to above.

Other than as permitted in any relevant BMJ Author's Self Archiving Policies, I confirm this Work has not been accepted for publication elsewhere, is not being considered for publication elsewhere and does not duplicate material already published. I confirm all authors consent to publication of this Work and authorise the granting of this licence.

For peer review only

Health impact of monoclonal gammopathy of undetermined significance (MGUS) and monoclonal B-cell lymphocytosis (MBL): findings from a UK population-based cohort

Corresponding author: Eve Roman, Epidemiology & Cancer Statistics Group, Department of Health Sciences, Seebohm Rowntree Building, University of York, YO10 5DX, UK: eve.roman@york.ac.uk

Maxine Lamb[1], Alexandra G Smith[1], Daniel Painter[1], Eleanor Kane[1], Tim Bagguley[1], Robert Newton[1,2], Debra Howell[1], Gordon Cook[3], Ruth de Tute[4], Andrew Rawstron[4], Russell Patmore[5], Eve Roman[1]

[1] Department of Health Sciences, University of York, YO10 5DD, UK

[2] Ugandan Virus Research Institute, Entebbe, Uganda

[3] Department of Haematology, Leeds Cancer Centre, St James's University Hospital, LS9 7LP, UK

[4] Haematological Malignancy Diagnostic Service, Leeds Cancer Centre, St James's University Hospital, LS9 7LP, UK

[5] Queens Centre for Oncology and Haematology, Hull University Teaching Hospitals NHS Trust, HU16 5JQ, UK

For peer review only

For peer review only - http://bmjopen.bmj.com/site/about/guidelines.xhtml

ABSTRACT

Objective: To examine mortality and morbidity patterns before and after premalignancy diagnosis in individuals with monoclonal gammopathy of undetermined significance (MGUS) and monoclonal B-cell lymphocytosis (MBL); and compare their secondary healthcare activity to that of the general population.

Design: Population-based patient cohort, within which each patient is matched at diagnosis to 10 age and sex-matched individuals from the general population. Both cohorts are linked to nationwide information on deaths, cancer registrations, and Hospital Episode Statistics (HES).

Setting: The UK's Haematological Malignancy Research Network; which has a catchment population of around 4 million served by 14 hospitals and a central diagnostic laboratory.

Participants: All patients newly diagnosed 2009-15 with MGUS (n=2,193) or MBL (n=561), and their age and sex-matched comparators (n= 27,538).

Main Outcome measures: Mortality, and hospital inpatient and outpatient activity in the five years before, and three years after, diagnosis.

Results: Individuals with MGUS experienced excess morbidity in the 5-years before diagnosis, and excess mortality and morbidity in the 3-years after. Increased rate ratios (RR) were evident for nearly all clinical specialties; the largest, both before and after diagnosis, being for nephrology (before RR=4.29, 95% Confidence Interval 3.90-4.71; after RR=13.8, 95% CI 12.8-15.0) and rheumatology (before RR=3.40, 95% CI 3.18-3.63; after RR=5.44, 95% CI 5.08-5.83). Strong effects were also evident for endocrinology, neurology, dermatology and respiratory medicine. Conversely, only marginal increases in mortality and morbidity were evident for MBL.

Conclusions: MGUS and MBL are generally considered to be relatively benign, since most individuals with monoclonal immunoglobulins never develop a B-cell malignancy or any other M-protein related organ/tissue related disorder. Nonetheless, our findings offer strong support for the view that in some individuals monoclonal gammopathy has the potential to cause systemic disease resulting in wide-ranging organ/tissue damage and excess mortality.

For peer review only

ARTICLE SUMMARY

Strengths and limitations of this study

- Data are from an established population-based cohort within which all haematological malignancies and related clonal disorders are diagnosed, monitored, and coded at a single laboratory.
- Providing nationally generalizable data, all diagnoses are included, and complete follow-up is achieved via linkage to nationwide administrative datasets.
- The age and sex-matched general population cohort enables baseline activity and rate ratios to be calculated, both before and after premalignancy detection.
- Analyses are constrained by the fact that hospital episodes statistics are primarily collected for administrative and clinical purposes, and not for research.

Funding

This work was supported by Cancer Research UK, grant numbers 18362 and 29685; and Blood Cancer UK, grant number 15037

Competing interests

None

Data sharing agreement

Ethical approvals and data restrictions mean that data cannot be shared; but collaborative projects can be undertaken. The corresponding author can be contacted for more information.

For peer review only

BACKGROUND

Monoclonal gammopathy of undetermined significance (MGUS) and monoclonal B-cell lymphocytosis (MBL) are premalignant monoclonal B-cell disorders; the former progressing to myeloma at a rate of around 1% per year[1,2] and the latter to chronic lymphocytic leukaemia (CLL) at around 2% per year.[3,4] Diagnosed more frequently in men than women and people over 60 years of age,[5,6] overt symptoms of haematological malignancy are, by definition, absent in both MBL and MGUS.[7] Accordingly, although some premalignant disorders are found coincidentally during routine health checks, others are identified during diagnostic work-up investigations; MGUS during the course of tests applied to detect a range of potential conditions and illnesses,[8,9] and MBL during episodes of unexplained lymphocytosis.[4,10]

In addition to the association with haematological malignancy, individuals with MGUS or MBL sometimes experience higher than expected levels of mortality and morbidity that are independent of cancer.[4,8,11–16] Indeed, although most individuals with these disorders suffer no obvious ill effects, interest in their relationship with other co-morbidities has increased markedly in recent years; MBL largely in relation to its potential to impact on the immune response,[17] and MGUS due to the systemic organ and tissue damage that can be caused by monoclonal immunoglobins secreted by the abnormal B-cell clone.[18] Hitherto, however, most information about these associations has been derived either from case-control studies established to look at risk factors for disease development (e.g. family history of disease), additional tests applied to specific patient groups (e.g. patients with kidney disease), or cohort studies that track individuals with either MGUS/MBL forwards in time from their diagnosis.[5,13,18] However, despite the undoubted interest in the sequence of health events, as far as we are aware no systematic population-based investigations of the co-morbidity patterns that precede and succeed a diagnosis of either MGUS or MBL have been undertaken in the same cohort.

With a view to shedding light on the health events occurring before and after the diagnosis of MGUS and MBL, the present report uses data from an established UK population-based patient cohort of haematological malignancies and related disorders to examine the co-morbidity patterns of individuals with these premalignancy clonal disorders (MGUS=2203, MBL=561). To enable effect size quantification, these patterns are compared to the baseline activity of an individually age- and sex-matched (10 per patient) general population comparison cohort.

For peer review only - http://bmjopen.bmj.com/site/about/guidelines.xhtml

For peer review only

METHODS

Cases are from the Haematological Malignancy Research Network (HMRN; www.HMRN.org), a specialist UK registry established in 2004 to provide robust generalizable data to inform contemporary clinical practice and research across the country as a whole.[19,20] HMRN operates under a legal basis that permits data to be collected directly from health records without explicit consent. Set within a catchment population of around four million that is served by 14 hospitals and has a socioeconomic profile which is broadly representative of the UK as a whole, all haematological cancers and related conditions are diagnosed and coded by clinical specialists at a single integrated haematopathology laboratory, the Haematological Malignancy Diagnostic Service (www.HMDS.info), using standardized diagnostic criteria and the latest WHO ICD-O-3 classification.[7] Specifically, in relation to the present report, which covers diagnoses made in 2009-15, MBL was defined by a peripheral blood monoclonal B-cell count <5 x10^9/L in individuals with no other features of a B-cell lymphoproliferative disorder; and MGUS by a serum paraprotein less than 30 g/L, and in those where a bone marrow examination was considered necessary following clinical examination, a clonal bone marrow plasma cells/lymphoplasmacytic infiltration of less than 10%. Hence, within the HMRN region, as in other diagnostic settings [21,22], invasive bone marrow examinations in MGUS patients are generally only carried when laboratory or clinical features are indicative of an underlying plasma cell neoplasm, lymphoproliferative disorder, monoclonal immunoglobulin deposition disease (e.g. amyloidosis), or conditions like POEMS syndrome. [7]

To facilitate comparisons with unaffected individuals, HMRN also has a general population cohort. To create this 'control' cohort, all patients diagnosed 2009-15 with a haematological malignancy or related clonal disorder (n=18,127) were individually matched on sex and age at the point of diagnosis to 10 randomly selected individuals from the same catchment population. All controls were assigned a serial number that linked them to their matched case, and a "pseudo-diagnosis" date that corresponded to their matched case's diagnosis date. Individuals in the patient cohort and the comparison cohort are linked to the same nationwide information on deaths, cancer registrations and Hospital Episode Statistics (HES). At the point of selection and matching, all controls were resident in the HMRN region and none had a previous cancer registration for a haematological malignancy. [23,24]. Hence for the control cohort (in contrast to the patient cohort) no additional health information outside that contained within national administrative datasets was available.

For peer review only - http://bmjopen.bmj.com/site/about/guidelines.xhtml

Using similar methods to those previously described,[23–25] associations with hospital inpatient activity (HES Admitted Patient Care; HES-APC) and outpatient activity (HES Outpatient; HES-OP) in the five years prior to diagnosis/pseudo-diagnosis through to the three years after diagnosis were investigated. HES inpatient data contains ICD-10 (International Statistical Classification of Diseases 10th revision) codes derived from discharge summaries[26]; and associations with these were examined in relation to the 17 specific conditions in the Charlson Comorbidity Index.[27–29] By contrast, HES outpatient data contains details about the type of outpatient attendance; the majority being linked to consultant specialty codes (e.g. ophthalmology, rheumatology etc.), with the remainder largely comprising routine follow-up/monitoring, nurse-led clinic attendances (e.g. anticoagulant clinics) and consultations with allied health professionals (e.g. podiatry).

This report includes all patients (cases) who were newly diagnosed with either MGUS (n=2193) or MBL (n=561) between 1st January 2009 and 31st December 2015 and their matched controls (n=27,538); individuals diagnosed with a haematological cancer within six months of their MGUS/MBL diagnosis were considered ineligible. All cases and controls were followed-up for cancer registration and death until March 2017, and hospital activity (inpatient and outpatient) until March 2016. Additionally, progressions and/or transformations among cases were identified through HMDS up to March 2017. Data were summarised using standard methods. Overall survival, hospital activity and rate ratios (RRs) were calculated using time-to-event analyses. The Stata programme ‘strel’ was used to estimate relative survival (RS), using age- and sex-specific background mortality rates from national life tables.[30,31] All analyses were conducted using Stata 16.0.

Patient and public involvement (PPI)

PPI is integral to HMRN, and takes place via a dedicated Patient Partnership, overseen by a lay committee. Patients from the partnership are involved in identifying key research questions and participate in all our funding applications. Furthermore, patients and their relatives routinely take part in the dissemination of HMRN’s findings, which also occurs via our project website: www.yhhn.org

RESULTS

Characteristics of individuals with either MGUS or MBL are summarized alongside their corresponding controls in Table 1. Both MGUS and MBL were more commonly diagnosed in males (51.6% MGUS, 58.5% MBL) and individuals over the age of 70 years (median diagnostic age 73.4

For peer review only - http://bmjopen.bmj.com/site/about/guidelines.xhtml

years for MGUS and 72.1 years for MBL). At 90.2% (95% Confidence Interval 87.4-92.4%), the 5-year relative survival (RS) of patients diagnosed with MGUS was significantly lower ($p<0.01$) than that of their general population controls (RS 99.5%, 95% CI 98.7-99.8%). For MBL, the difference was far less marked; the 5-year RS being 94.3% (95% CI 86.3-97.7%) for MBL cases and 98.5% (95% CI 96.8-99.3%) for their controls. Interestingly, the observed variations in blood cancer progression were in the opposite direction to those seen for mortality: 75 (3.4%) MGUS patients were diagnosed with a haematological malignancy (48/75 were myelomas) before April 2017, compared to 140 (25.0%) MBL patients (137/140 were CLLs). No significant differences were, however, evident for non-haematological cancer registrations; although the frequencies were slightly higher for MGUS (8.4%) and MBL (10.3%) than for their corresponding controls (8.0% and 8.8% respectively).

For peer review only

For peer review only - http://bmjopen.bmj.com/site/about/guidelines.xhtml

Table 1: Characteristics of patients diagnosed with monoclonal gammopathy of undetermined significance (MGUS) or monoclonal B-cell lymphocytosis (MBL) and their corresponding controls; HMRN diagnoses 2009-2015

		MGUS		MBL	
		Cases Number (%)	Controls Number (%)	Cases Number (%)	Controls Number (%)
**Total**		2193 (100)	21928 (100)	561 (100)	5610 (100)
**Gender**	**Male**	1131 (51.6)	10620 (48.4)	328 (58.5)	3280 (58.5)
	**Female**	1062 (48.4)	11308 (51.6)	233 (41.5)	2330 (41.5)
**Age (years)**	**<60**	347 (15.8)	3470 (15.8)	85 (15.2)	850 (15.2)
	**60-70**	500 (22.8)	5000 (22.8)	153 (27.3)	1530 (27.3)
	**70-80**	782 (35.7)	7820 (35.7)	194 (34.6)	1940 (34.6)
	**≥80**	564 (25.7)	5638 (25.7)	129 (23.0)	1290 (23.0)
	**Median (IQR)**	73.4 (64.6 - 80.2)	73.4 (64.6 - 80.2)	72.1 (64.5 - 79.2)	72.1 (64.5 - 79.2)
**Overall survival[1]**	**5-year (95% CI)**	71.9 (69.5-74.1)	80.1 (79.4-80.7)	77.2 (72.5-81.2)	80.7 (79.4-81.9)
**Relative survival**	**5-year (95% CI)**	90.2 (87.4-92.4)	99.5 (98.7-99.8)	94.3 (86.3, 97.7)	98.5 (96.8-99.3)
**Progressions and Transformations[1]**	**Total**	75 (3.4)		140 (25.0)	
	**Myeloma**	48 (2.2)	-	-	-
	**CLL[2]**	1 (0.05)	-	137 (24.4)	-
	**Other**	26 (1.2)	-	3 (0.5)	-
**Non-haematological cancer registrations[1]**	**Total**	185 (8.4)	1748 (8.0)	58 (10.3)	494 (8.8)
	**Lung**	31 (1.5)	190 (0.9)	4 (0.7)	59 (1.1)
	**Prostate[3]**	20 (1.8)	178 (1.6)	3 (0.9)	56 (1.7)
	**Colorectal**	19 (0.9)	166 (0.8)	6 (1.1)	40 (0.7)
	**Breast[3]**	15 (1.4)	135 (1.3)	4 (1.7)	16 (0.7)
**Comorbidity score before MGUS/MBL diagnosis[4]**	**0**	1256 (57.3)	15893 (72.5)	419 (74.7)	4115 (73.4)
	**1**	506 (23.1)	3617 (16.5)	84 (15.0)	898 (16.0)
	**≥2**	431 (19.7)	2418 (11.0)	58 (10.3)	597 (10.6)
		p<0.001		p=0.78	

[1]Followed-up until 15/03/2017; [2]Chronic lymphocytic leukaemia; [3]Prostate for men only, breast for women only; [4]≥1 hospital admission with a diagnosis of one the 17 conditions included in the Charlson Comorbidity Index[25-27] during the 5 years to 1 month before MGUS/MBL diagnosis (patients)/pseudo-diagnosis (controls)

With respect to co-morbidity, in the years leading up to diagnosis MGUS patients were significantly more likely than their corresponding controls to have a record of at least one of the 17 co-morbidities specified in the Charlson Comorbidity Index[27–29] recorded in their discharge summaries; but no differences between MBL cases and their controls were evident (Table 1). More information about hospital activity patterns of cases with MGUS/MBL and their general population controls, is shown in Figure 1; which shows inpatient and outpatient activity (excluding

For peer review only - http://bmjopen.bmj.com/site/about/guidelines.xhtml

For peer review only

haematology) during the five-years before and the three-years after diagnosis of MGUS (Figure 1A) and MBL (Figure 1B). In the period before diagnosis, patients with MGUS (Figure 1A) had consistently higher outpatient activity rates than their controls, the disparity increasing markedly during the 18 months leading up to the formal diagnosis of MGUS by haematopathology where it remained high for about 12 months, before gradually falling and levelling out at a higher level than before diagnosis. Although less pronounced, a similar pattern is evident in inpatient data. With smaller numbers and more scatter, variations in outpatient and inpatient activity in MBL are less evident (Figure 1B).

Figure 2 shows outpatient attendance frequencies (at least two specialty-specific visits) in the three years before and in the three years after MGUS diagnosis for the top 25 clinical specialties; visits within one month (±) of diagnosis/pseudodiagnosis are excluded. As is evident from the plot, the increased outpatient activity seen among cases (Figure 1) occurs across a range of clinical specialties; the highest frequencies occurring in ophthalmology, haematology, general surgery, orthopaedics, general (internal) medicine and rheumatology. However, excluding haematology where, as expected, attendances increased markedly just before and after MGUS diagnosis, the largest rate ratios (RR) both before (Figure 3A) and after (Figures 3B) diagnosis were for nephrology (before diagnosis RR= 4.29, 95% CI 3.90-4.71; after diagnosis RR= 13.8, 95% CI 12.8-15.0) and rheumatology (before diagnosis RR= 3.40, 95% CI 3.18-3.63; after diagnosis RR= 5.44, 95% CI 5.08-5.83). Other significant associations ($p<0.05$) with rate ratio point estimates above 2.0 were evident for endocrinology, neurology, and respiratory medicine, as well as for the nurse-led monitoring activities which form part of on-going clinical care across a range of specialties.

MGUS data are stratified by subtype in Table 2. Accounting for around two-thirds of the total (n=1471; 67.0%) the IgG subtype dominates, followed by IgM (n=350; 16.0%) and IgA (n=266; 12.1%). The remaining 106 (4.8%) 'other' category comprise a mix of subtypes: light chain only (n=60), IgG + IgM (n=17), IgG + IgA (n=6), IgA + IgM (n=1), IgE (n=2); and not recorded (n=20). As expected, progression to myeloma in the three years following MGUS diagnosis was largely restricted to the IgG and IgA subtypes. The age distributions, five-year survival estimates (overall and relative) and non-haematological malignancy frequencies of the main subtypes were broadly similar; although patients in the combined 'other' category tended to be slightly older and to fare less well (RS= 77.6%, 95% CI 62.6-87.2%). The numbers of patients in the individual groups were, however, too sparse to examine the data in greater depth. Finally, during the study period (2009-15), in addition to the detection of a paraprotein in peripheral blood, around 70% (1527/2193) of MGUS

For peer review only - http://bmjopen.bmj.com/site/about/guidelines.xhtml

patients in our cohort had a confirmatory bone marrow examination taken to exclude an underlying neoplasm. The patient characteristics and secondary care activity patterns of those who had bone marrow examinations were, however, broadly similar to those who did not (data not shown).

For peer review only

For peer review only - http://bmjopen.bmj.com/site/about/guidelines.xhtml

Table 2: Characteristics of patients diagnosed with monoclonal gammopathy of undetermined significance (MGUS) and their corresponding controls, stratified by MGUS subtype; HMRN diagnoses 2009-2015

	MGUS subtype							
	IgG		IgM		IgA		Other[1]	
	Cases Number (%)	Controls Number (%)	Cases Number (%)	Controls Number (%)	Cases Number (%)	Controls Number (%)	Cases Number (%)	Controls Number (%)
**Total**	1471 (100.0)	14709 (100.0)	350 (100.0)	3499 (100.0	266 (100.0)	2660 (100.0)	106 (100.0)	1060 (100.0)
**Gender**								
**Male**	745 (50.6)	7449 (50.6)	177 (50.6)	1769 (50.6)	145 (54.5)	1450 (54.5)	64 (60.4)	640 (60.4)
**Female**	726 (49.4)	7260 (49.4)	173 (49.4)	1730 (49.4)	121 (45.5)	1210 (45.5)	42 (39.6)	420 (39.6)
**Age (years)**								
**<60**	239 (16.2)	2390 (16.2)	50 (14.3)	500 (14.3)	48 (18.0)	480 (18.0)	10 (9.4)	100 (9.4)
**60-70**	336 (22.8)	3360 (22.8)	80 (22.9)	800 (22.9)	56 (21.1)	560 (21.1)	28 (26.4)	280 (26.4)
**70-80**	514 (34.9)	5140 (34.9)	130 (37.1)	1300 (37.2)	104 (39.1)	1040 (39.1)	34 (32.1)	340 (32.1)
**≥80**	382 (26.0)	3819 (26.0)	90 (25.7)	899 (25.7)	58 (21.8)	580 (21.8)	34 (32.1)	340 (32.1)
**Median**	73.4	73.4	73.0	73.0	73.0	73.0	75.3	75.3
**Survival 5-year (95% CI)[2]**								
**Overall**	72.3 (69.4-74.9)	79.5 (78.7-80.3)	69.6 (63.3-75.0)	81.3 (79.7-82.9)	74.2 (67.3-79.8)	82.4 (80.6-84.1)	68.6 (56.5-78.0)	77.6 (74.1-80.7)
**Relative**	90.6 (86.9-93.2)	99.2 (98.3-99.7)	84.2 (75.8-89.8)	99.3 (93.7-99.9)	88.3 (78.5-93.8)	98.5 (95.3-99.5)	77.6 (62.6-87.2)	96.8 (89.5-99.1)
**Progressions/transformations[2]**								
**Total**	44 (3.0)	-	8 (2.3)	-	17 (6.4)	-	6 (5.7)	-
**Myeloma**	34 (2.3)	-	1 (0.3)	-	11 (4.1)	-	2 (1.9)	-
**Other**	10 (0.7)	-	7 (2.0)	-	6 (2.3)	-	4 (3.8)	-
**Non-haematological cancer registrations[2]**								
**Total**	118 (8.0)	1173(8.0)	41 (11.7)	277 (7.9)	19 (7.1)	210 (7.9)	7 (6.6)	88 (8.4)
**Lung**	16 (1.1)	133 (0.9)	6 (1.7)	24 (0.7)	4 (1.5)	21 (0.8)	5 (4.7)	12 (1.1)
**Prostate[3]**	14 (1.9)	115 (1.5)	3 (1.7)	27 (1.5)	3 (2.1)	25 (1.7)	-	11 (1.7)
**Colorectal**	16 (1.1)	112 (0.8)	2 (0.6)	26 (0.7)	1 (0.4)	20 (0.7)	-	8 (0.8)
**Breast[3]**	9 (1.2)	88 (1.2)	4 (2.3)	26 (1.5)	2 (1.7)	15 (1.2)	-	6 (1.4)
**Hospital outpatient activity (≥ 3 visits), excluding haematology and visits within 1 month of diagnosis[4]**								
**5-years before diagnosis**	1173 (79.7)	9103 (61.9)	287 (82.0)	2155 (61.6)	216 (81.2)	1633 (61.4)	89 (84.0)	663 (62.5)
**3-years after diagnosis**	964 (65.5)	6558 (44.6)	240 (68.6)	1577 (45.1)	176 (66.2)	1154 (43.4)	70 (66.0)	445 (42.0)

[1]Biclonal, IgD, IgE, light chain, not known; [2]Followed-up until 15/03/2017; [3]Prostate for men only, breast for women only; [4]In the 5 years before diagnosis/pseudo-diagnosis and 3 years after diagnosis/pseudo-diagnosis

For peer review only - http://bmjopen.bmj.com/site/about/guidelines.xhtml

DISCUSSION

Including data on nearly 3000 cases with pre-malignant clonal disorders and ten times as many age and sex-matched general population controls, this large UK record-linkage study found that individuals with MGUS not only experienced excess mortality and morbidity after diagnosis, but also excess morbidity in the 5 years before. By contrast, only marginal increases in mortality and morbidity were evident for MBL; none of which were consistent or varied significantly from the general population. Interestingly, progression patterns were in the opposite direction: in the years following detection of a pre-malignant clonal disorder, 3.4% (n=75/2193) of those with MGUS developed a haematological malignancy (41 of which were myelomas) before April 2017, compared to 25.0% (n=140/561) of those with MBL (137 of which were CLLs).

The elevated mortality and morbidity following a diagnosis of MGUS, which seems largely independent of progression to cancer, is consistent with reports relating to the potential clinical significance of this disorder.[11–18] Corresponding to the period of diagnostic work-up, our data also demonstrate the pronounced increase in hospital activity in the months surrounding MGUS diagnosis; the highest activity being observed in the six months before and six months after diagnosis. Of more importance, perhaps, the analyses clearly show that hospital activity in people subsequently diagnosed with MGUS is often elevated many years before diagnosis: excesses being observed in specialties covering most organ and tissue systems including nephrology, endocrinology, neurology, rheumatology, gastroenterology, dermatology and respiratory medicine. By contrast, although hospital activity increased in the months around the time of MBL diagnosis, no consistent differences or patterns either before or after then, were detected. Furthermore, in agreement with findings reported in other studies that used age and sex-matched controls, no associations with mortality were detected.[3,32] However, given the fact that MBL has been associated with increased susceptibility to infection and non-CLL related mortality [4,15,33], it is possible that the findings relating to subsequent morbidity and mortality could change as our data mature, length of follow-up increases, and linkage to primary care data becomes possible.

The age and sex distributions of our population-based cohorts are broadly similar to those of other published MBL [3,34,35] and MGUS [1,2,11] series; as is the dominance of the IgG MGUS subtype.[1,2] Providing nationally generalizable data, additional strengths of our study include its large well-defined population, within which all haematological malignancies and related clonal disorders are diagnosed, monitored, and coded using up-to-date standardized procedures at a central haematopathology laboratory.[19] In this context, it is important to bear in mind that most people with premalignant clonal disorders remain asymptomatic, and that our cohorts contain a relatively large proportion of people who came to clinical attention in primary and/or secondary care and were referred to haematology for further investigation. More specifically, around 98% of patients in our MBL cohort had high-count MBL, and within the HMRN region MBL patients are monitored routinely using flow cytometry so laboratory progressions

For peer review only

(B-cell count >5 x10^9/L) may be detected with higher sensitivity than in cases monitored clinically. This is supported by the fact that over the follow-up period (median 3.8 years), around a quarter of MBL patients progressed to CLL, and 3.6% required treatment. Prevalence comparisons with population screening studies also confirm that the majority of those over 50 years of age with monoclonal immunoglobulin in their blood/urine would not be included in our MGUS cohort.[5,20,35–37] In this context, it is important to remember that some members of the control cohort would, if screened, have had a premalignant clonal disorder detected in their peripheral blood. [13,32,33,38,39] Unfortunately, however, information on diagnostic work-up tests and monitoring procedures is not routinely included in nationally compiled HES. Furthermore, the anonymised nature of the control cohort means that individuals cannot be linked to other data sources. Hence, although we know that members of the control cohort had no prior record of an MGUS or MBL diagnosis within the study region in the years leading up to their corresponding case's diagnosis, we do not know how many people developed these conditions after their case was diagnosed.

The diversity of morbidity effects seen among individuals with MGUS is consistent with the expanding body of evidence relating to the potential adverse impact that even low levels of circulating monoclonal protein (M-protein) can have. Thus far, the complex underpinning mechanisms identified include; deposition of M-protein aggregations of varying immunoglobulin subtypes in different organs, as well as the induction of auto-antibodies and cytokines that can impact on organs and tissues in a variety of deleterious ways.[13,18,40,41] Indeed, the recognized number of M-protein mediated entities is increasing, with several affecting multiple organs; well-known deposition syndromes including primary amyloidosis and paraneoplastic conditions such as POEMS syndrome (polyneuropathy, organomegaly, endocrinopathy, monoclonal gammopathy, and skin changes).[7,42] As evidenced in our analysis, kidney involvement is frequent, both in the years before (four-fold excess) and after (fourteen-fold excess) MGUS diagnosis. Indeed, the umbrella term monoclonal gammopathy of renal significance (MGRS) has recently been suggested to cover all M-protein mediated kidney disorders that fail to meet the diagnostic criteria for multiple myeloma or any other B-cell malignancy.[13,18,43] Other organ-specific terms continue to emerge, and with a view to improving recognition of these complex disorders which clearly pose significant diagnostic and treatment challenges, the overarching term monoclonal gammopathy of clinical significance (MGCS) has also been suggested.[18]

From a haematological malignancy perspective, MGUS and MBL are generally considered to be relatively benign conditions. However, both can have other deleterious health consequences; the effect of monoclonal gammopathy being particularly striking. Impacting significantly on survival and having the potential to cause systemic disease and wide-ranging damage to most organs and tissues, the adverse outcomes associated with the M-proteins produced by the abnormal B-cell clone can be severe and extend over many years. Even though most people with monoclonal immunoglobulins never develop a B-cell malignancy or suffer from any other form of M-protein related organ/tissue related disorder, the

For peer review only

consequences for those that do can be extremely serious. In this regard, early targeting of pathogenic B-cell clones could mitigate both cancer and non-cancer effects; but currently, although knowledge is increasing, there is no known way to reliably identify such clones in the absence of other signs/symptoms. Hence, population screening cannot be recommended, and diagnosis remains reliant on clinical suspicion. However, the long-standing nature of the co-morbidity associations seen prior to MGUS diagnosis in our data, suggest that there may be room for improvement, and that the implementation of strategies to improve awareness and earlier detection, as well as monitoring of high-risk patient groups, could prove beneficial.

Ethics Approval: The Haematological Malignancy Research Network has ethics approval (REC 04/01205/69) from Leeds West Ethics Committee, R&D approval from each NHS Trust, and exemption from Section 251 of the Health & Social Care Act (PIAG 1-05 9h)/2007)

Contributors: ER, AS, DH and RP initiated the patient cohort within which this research is nested; and ER, AS, and EK designed the comparison cohort. ER, AS and ML planned this study and analyses. R de T and AR oversaw all laboratory procedures; and ML, DP, EK, TB managed the data and carried out data analysis. GC, RP, AR, and RN commented on the biological and clinical aspects. ER and ML drafted the manuscript, which was approved by all authors.

References

1 Kyle RA, Larson DR, Therneau TM, *et al.* Long-Term Follow-up of Monoclonal Gammopathy of Undetermined Significance. *N Engl J Med* 2018;**378**:241–9. doi:10.1056/NEJMoa1709974

2 Turesson I, Kovalchik SA, Pfeiffer RM, *et al.* Monoclonal gammopathy of undetermined significance and risk of lymphoid and myeloid malignancies: 728 cases followed up to 30 years in Sweden. *Blood* 2014;**123**:338–45. doi:10.1182/blood-2013-05-505487

3 Parikh SA, Chaffee KG, Larson MC, *et al.* Outcomes of a large cohort of individuals with clinically ascertained high-count monoclonal B-cell lymphocytosis. *Haematologica* 2018;**103**:e237–40. doi:10.3324/haematol.2017.183194

4 Rawstron AC, Bennett FL, O'Connor SJM, *et al.* Monoclonal B-cell lymphocytosis and chronic lymphocytic leukemia. *N Engl J Med* 2008;**359**:575–83. doi:10.1056/NEJMoa075290

5 Goldin LR, McMaster ML, Caporaso NE. Precursors to lymphoproliferative malignancies. *Cancer Epidemiol Biomarkers Prev* 2013;**22**:533–9. doi:10.1158/1055-9965.EPI-12-1348

6 Smith A, Howell D, Patmore R, *et al.* Incidence of haematological malignancy by sub-type: a report from the Haematological Malignancy Research Network. *Br J Cancer* 2011;**105**:1684–92. doi:10.1038/bjc.2011.450

7 Swerdlow S, Campo E, Harris N, *et al. WHO Classification of Tumours of Haematopoietic and Lymphoid Tissues*. Lyon, France: : World Health Organization 2017.

For peer review only

8 Kristinsson SY, Björkholm M, Andersson TM-L, *et al.* Patterns of survival and causes of death following a diagnosis of monoclonal gammopathy of undetermined significance: a population-based study. *Haematologica* 2009;**94**:1714–20. doi:10.3324/haematol.2009.010066

9 Merlini G, Palladini G. Differential diagnosis of monoclonal gammopathy of undetermined significance. *Hematology Am Soc Hematol Educ Program* 2012;**2012**:595–603. doi:10.1182/asheducation.V2012.1.595.3798563

10 Shanafelt TD, Ghia P, Lanasa MC, *et al.* Monoclonal B-cell lymphocytosis (MBL): biology, natural history and clinical management. *Leukemia* 2010;**24**:512–20. doi:10.1038/leu.2009.287

11 Kristinsson SY, Tang M, Pfeiffer RM, *et al.* Monoclonal gammopathy of undetermined significance and risk of infections: a population-based study. *Haematologica* 2012;**97**:854–8.

12 Thorsteinsdottir S, Lund SH, Lindqvist EK, *et al.* Bone disease in monoclonal gammopathy of undetermined significance: results from a screened population-based study. *Blood Adv* 2017;**1**:2790–8. doi:10.1182/bloodadvances.2017010454

13 Glavey SV, Leung N. Monoclonal gammopathy: The good, the bad and the ugly. *Blood Rev* 2016;**30**:223–31. doi:10.1016/j.blre.2015.12.001

14 Ciocchini M, Arbelbide J, Musso CG. Monoclonal gammopathy of renal significance (MGRS): the characteristics and significance of a new meta-entity. *Int Urol Nephrol* 2017;**49**:2171–5. doi:10.1007/s11255-017-1594-y

15 Moreira J, Rabe KG, Cerhan JR, *et al.* Infectious complications among individuals with clinical monoclonal B-cell lymphocytosis (MBL): a cohort study of newly diagnosed cases compared to controls. *Leukemia* 2013;**27**:136–41.

16 Rögnvaldsson S, Steingrímsson V, Turesson I, *et al.* Peripheral neuropathy and monoclonal gammopathy of undetermined significance: a population-based study including 15,351 cases and 58,619 matched controls. *Haematologica* Published Online First: 13 February 2020. doi:10.3324/haematol.2019.239632

17 Criado I, Blanco E, Rodríguez-Caballero A, *et al.* Residual normal B-cell profiles in monoclonal B-cell lymphocytosis versus chronic lymphocytic leukemia. *Leukemia* 2018;**32**:2701–5. doi:10.1038/s41375-018-0164-3

18 Fermand J-P, Bridoux F, Dispenzieri A, *et al.* Monoclonal gammopathy of clinical significance: a novel concept with therapeutic implications. *Blood* 2018;**132**:1478–85. doi:10.1182/blood-2018-04-839480

19 Smith A, Howell D, Crouch S, *et al.* Cohort Profile: The Haematological Malignancy Research Network (HMRN); a UK population-based patient cohort. *Int J Epidemiol* Published Online First: 2018. doi:10.1093/ije/dyy044

20 Haematological Malignancy Research Network. HMRN - Statistics. https://www.hmrn.org/statistics (accessed 12 Apr 2020).

21 Kyle RA, Therneau TM, Rajkumar SV, *et al.* A long-term study of prognosis in monoclonal gammopathy of undetermined significance. *N Engl J Med* 2002;**346**:564–9. doi:10.1056/NEJMoa01133202

22 Landgren O, Kristinsson SY, Goldin LR, *et al.* Risk of plasma cell and lymphoproliferative disorders among 14621 first-degree relatives of 4458 patients with monoclonal gammopathy of undetermined significance in Sweden. *Blood* 2009;**114**:791–5. doi:10.1182/blood-2008-12-191676

For peer review only

23 Kane E, Painter D, Smith A, *et al.* The impact of rheumatological disorders on lymphomas and myeloma: a report on risk and survival from the UK's population-based Haematological Malignancy Research Network. *Cancer Epidemiology* 2019;**59**:236–43. doi:10.1016/j.canep.2019.02.014

24 Kane E, Painter D, Smith A, *et al.* Risk of mature B-cell neoplasms and precursor conditions after joint replacement: a report from the Haematological Malignancy Research Network. *Int J Cancer* Published Online First: 1 November 2019. doi:10.1002/ijc.32765

25 Lamb MJ, Roman E, Howell DA, *et al.* Hodgkin lymphoma detection and survival: findings from the Haematological Malignancy Research Network. *BJGP Open* 2019;**3**. doi:10.3399/bjgpopen19X101668

26 World Health Organization. International Statistical Classification of Diseases and Related Health Problems, 10th revision. 2010.

27 Maringe C, Fowler H, Rachet B, *et al.* Reproducibility, reliability and validity of population-based administrative health data for the assessment of cancer non-related comorbidities. *PLOS ONE* 2017;**12**:e0172814. doi:10.1371/journal.pone.0172814

28 Wästerlid T, Mohammadi M, Smedby KE, *et al.* Impact of comorbidity on disease characteristics, treatment intent and outcome in diffuse large B-cell lymphoma: a Swedish lymphoma register study. *Journal of Internal Medicine* 2019;**285**:455–68. doi:10.1111/joim.12849

29 Charlson ME, Pompei P, Ales KL, *et al.* A new method of classifying prognostic comorbidity in longitudinal studies: Development and validation. *Journal of Chronic Diseases* 1987;**40**:373–83. doi:10.1016/0021-9681(87)90171-8

30 Estève J, Benhamou E, Croasdale M, *et al.* Relative survival and the estimation of net survival: Elements for further discussion. *Statist Med* 1990;**9**:529–38. doi:10.1002/sim.4780090506

31 Cancer Research UK Cancer Survival Group. UK life tables. UK life tables. 2017.http://csg.lshtm.ac.uk/tools-analysis/uk-life-tables/ (accessed 26 Jan 2018).

32 Strati P, Shanafelt TD. Monoclonal B-cell lymphocytosis and early-stage chronic lymphocytic leukemia: diagnosis, natural history, and risk stratification. *Blood* 2015;**126**:454–62. doi:10.1182/blood-2015-02-585059

33 Criado I, Rodríguez-Caballero A, Gutiérrez ML, *et al.* Low-count monoclonal B-cell lymphocytosis persists after seven years of follow up and is associated with a poorer outcome. *Haematologica* 2018;**103**:1198–208. doi:10.3324/haematol.2017.183954

34 Rawstron AC, Shanafelt T, Lanasa MC, *et al.* Different biology and clinical outcome according to the absolute numbers of clonal B-cells in monoclonal B-cell lymphocytosis (MBL). *Cytometry B Clin Cytom* 2010;**78 Suppl 1**:S19-23. doi:10.1002/cyto.b.20533

35 Call TG, Norman AD, Hanson CA, *et al.* Incidence of chronic lymphocytic leukemia and high-count monoclonal B-cell lymphocytosis using the 2008 guidelines. *Cancer* 2014;**120**:2000–5. doi:10.1002/cncr.28690

36 Wadhera RK, Rajkumar SV. Prevalence of monoclonal gammopathy of undetermined significance: a systematic review. *Mayo Clin Proc* 2010;**85**:933–42. doi:10.4065/mcp.2010.0337

37 Li J, Smith A, Crouch S, *et al.* Estimating the prevalence of hematological malignancies and precursor conditions using data from Haematological Malignancy Research Network (HMRN). *Cancer Causes Control* 2016;**27**:1019–26. doi:10.1007/s10552-016-0780-z

For peer review only

38 Kyle RA, Therneau TM, Rajkumar SV, *et al.* Prevalence of monoclonal gammopathy of undetermined significance. *N Engl J Med* 2006;**354**:1362–9. doi:10.1056/NEJMoa054494

39 Landgren O, Graubard BI, Kumar S, *et al.* Prevalence of myeloma precursor state monoclonal gammopathy of undetermined significance in 12372 individuals 10-49 years old: a population-based study from the National Health and Nutrition Examination Survey. *Blood Cancer J* 2017;**7**:e618. doi:10.1038/bcj.2017.97

40 Merlini G, Stone MJ. Dangerous small B-cell clones. *Blood* 2006;**108**:2520–30. doi:10.1182/blood-2006-03-001164

41 Atkin C, Richter A, Sapey E. What is the significance of monoclonal gammopathy of undetermined significance? *Clin Med (Lond)* 2018;**18**:391–6. doi:10.7861/clinmedicine.18-5-391

42 Dispenzieri A. POEMS Syndrome: 2019 Update on diagnosis, risk-stratification, and management. *Am J Hematol* 2019;**94**:812–27. doi:10.1002/ajh.25495

43 Amaador K, Peeters H, Minnema MC, *et al.* Monoclonal gammopathy of renal significance (MGRS) histopathologic classification, diagnostic workup, and therapeutic options. *Neth J Med* 2019;**77**:243–54.

Figure Legends

Figure 1: Inpatient (IP) and outpatient (OP) monthly hospital visit activity rates five years before to three years after diagnosis (cases)/pseudo-diagnosis (controls) excluding haematology attendances: A) monoclonal gammopathy of undetermined significance (MGUS) and B) monoclonal B-cell lymphocytosis (MBL) diagnosed 2009-15

Figure 2: Percentage of cases and controls with at least two specialty-specific outpatient visits in the three years before and after diagnosis of monoclonal gammopathy of undetermined significance (MGUS). Top 25 recorded specialties, with visits within one month of diagnosis/pseudodiagnosis excluded

Figure 3: Monthly outpatient attendance rates (per 100 persons) in cases and controls and rate ratios by outpatient specialty with at least two visits (A) in the three years before diagnosis and (B) in the three years after diagnosis of monoclonal gammopathy of undetermined significance (MGUS)

For peer review only

Figure 1: Inpatient (IP) and outpatient (OP) monthly hospital visit activity rates five years before to three years after diagnosis (cases)/pseudo-diagnosis (controls) excluding haematology attendances: A) monoclonal gammopathy of undetermined significance (MGUS) and B) monoclonal B-cell lymphocytosis (MBL) diagnosed 2009-15

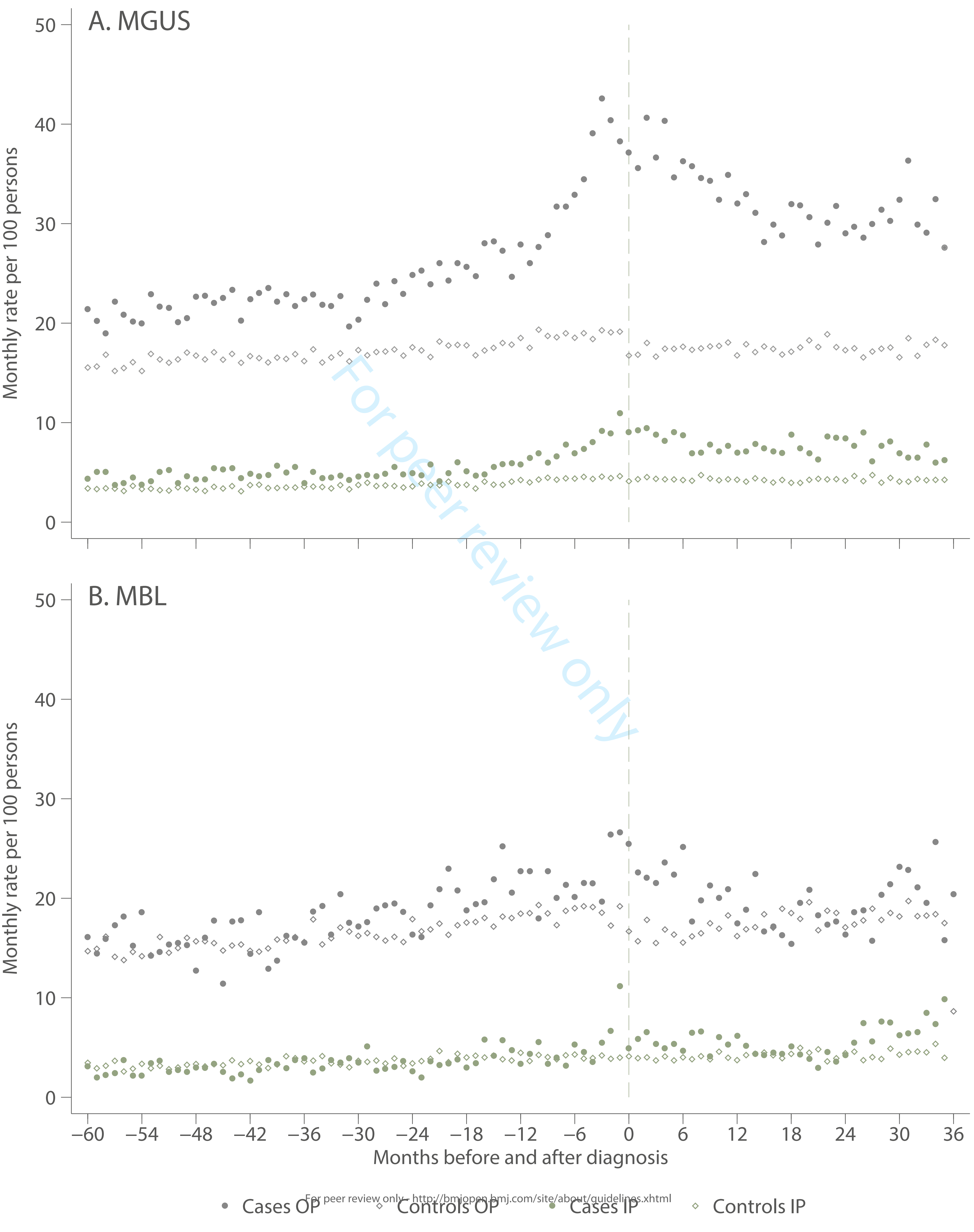

For peer review only

Figure 2: Percentage of cases and controls with at least two specialty-specific outpatient visits in the three years before and after diagnosis of monoclonal gammopathy of undetermined significance (MGUS). Top 25 recorded specialties, with visits within one month of diagnosis/pseudodiagnosis excluded.

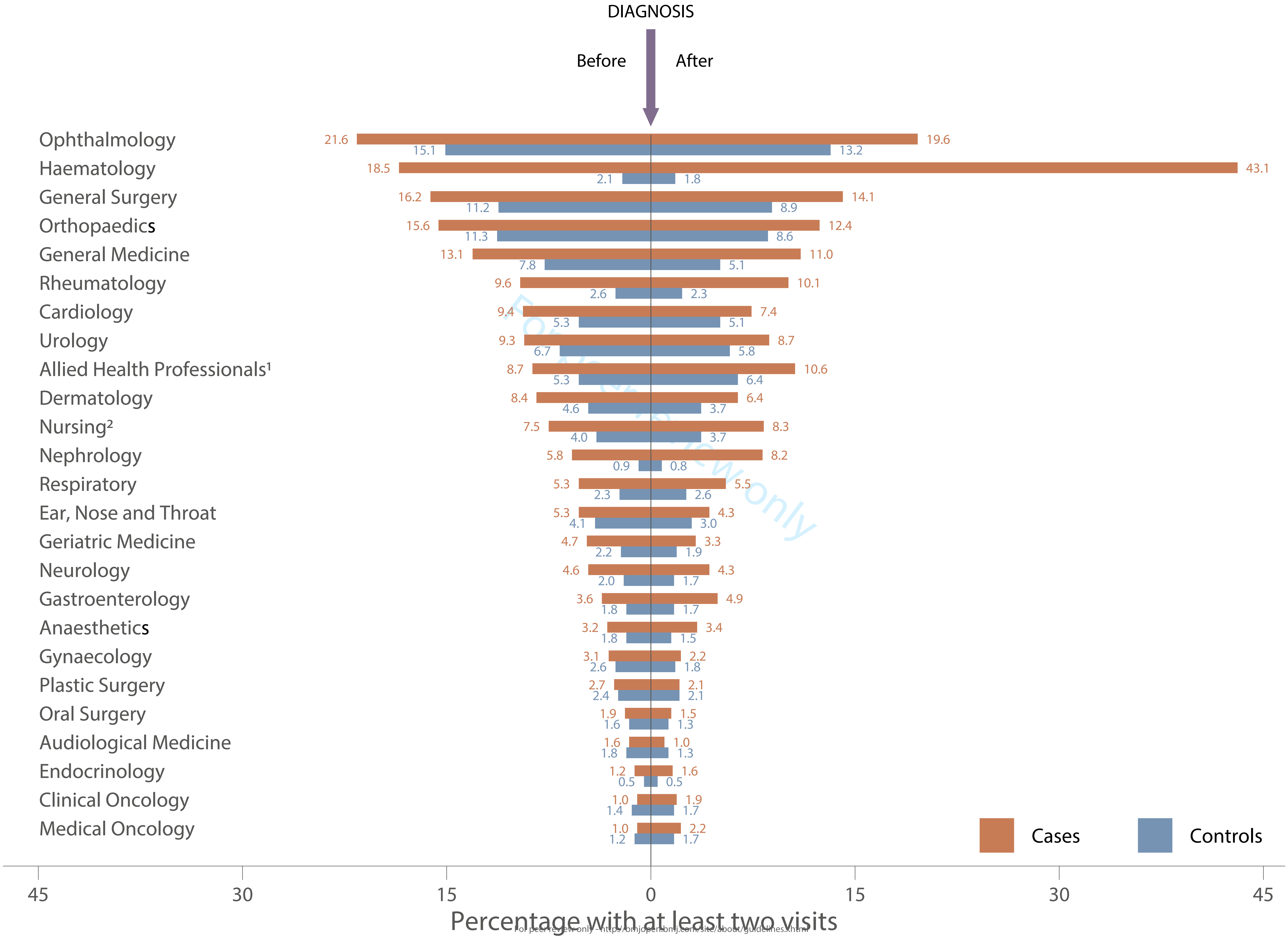

Figure 3: Monthly outpatient attendance rates (per 100 persons) in cases and controls and rate ratios by outpatient specialty with at least two visits (A) in the three years before diagnosis and (B) in the three years after diagnosis of monoclonal gammopathy of undetermined significance (MGUS)

Outpatient specialty	Post–diagnosis rank	Case rate	Control rate	Rate ratio (95% CI)
Nephrology	1	0.91	0.21	4.29 (3.90–4.71)
Rheumatology	2	1.70	0.50	3.40 (3.18–3.63)
Nursing	6	1.45	0.62	2.34 (2.19–2.51)
Endocrinology	4	0.15	0.07	2.19 (1.77–2.70)
Respiratory	8	0.74	0.35	2.11 (1.92–2.32)
Gastroenterology	3	0.40	0.21	1.90 (1.67–2.16)
Neurology	5	0.52	0.28	1.83 (1.63–2.05)
Dermatology	10	1.54	0.85	1.81 (1.70–1.93)
General Medicine	7	2.57	1.48	1.77 (1.67–1.87)
Allied Health Professionals	11	1.53	0.93	1.65 (1.55–1.76)
Geriatric Medicine	12	0.45	0.28	1.63 (1.44–1.83)
Cardiology	13	1.16	0.74	1.57 (1.46–1.69)
Anaesthetics	9	0.40	0.26	1.51 (1.33–1.71)
Ophthalmology	16	3.78	2.92	1.30 (1.25–1.35)
General Surgery	14	1.99	1.57	1.27 (1.20–1.34)
Orthopaedics	15	2.15	1.74	1.24 (1.17–1.31)
Urology	18	1.27	1.08	1.18 (1.10–1.26)
Ear, Nose and Throat	17	0.66	0.58	1.13 (1.02–1.24)
Plastic Surgery	20	0.42	0.40	1.07 (0.95–1.21)
Gynaecology	19	0.38	0.37	1.03 (0.90–1.16)

Outpatient specialty	Pre–diagnosis rank	Case rate	Control rate	Rate ratio (95% CI)
Nephrology	1	2.41	0.17	13.8 (12.8–15.0)
Rheumatology	2	2.07	0.39	5.44 (5.08–5.83)
Gastroenterology	6	0.61	0.20	3.03 (2.69–3.40)
Endocrinology	4	0.19	0.06	2.95 (2.39–3.65)
Neurology	7	0.66	0.25	2.64 (2.36–2.95)
Nursing	3	1.30	0.59	2.63 (2.45–2.82)
General Medicine	9	2.54	1.06	2.56 (2.41–2.72)
Respiratory	5	0.88	0.39	2.28 (2.07–2.50)
Anaesthetics	13	0.51	0.22	2.33 (2.06–2.65)
Dermatology	8	1.53	0.71	2.16 (2.01–2.32)
Allied Health Professionals	10	2.13	1.14	1.87 (1.76–1.98)
Geriatric Medicine	11	0.40	0.22	1.84 (1.60–2.11)
Cardiology	12	1.15	0.69	1.66 (1.53–1.80)
General Surgery	15	2.08	1.27	1.64 (1.54–1.74)
Orthopaedics	16	2.15	1.39	1.54 (1.45–1.64)
Ophthalmology	14	3.90	2.58	1.51 (1.44–1.58)
Ear, Nose and Throat	18	0.62	0.45	1.38 (1.24–1.54)
Urology	17	1.28	0.94	1.36 (1.26–1.47)
Gynaecology	20	0.37	0.29	1.27 (1.10–1.46)
Plastic Surgery	19	0.31	0.33	0.92 (0.79–1.07)

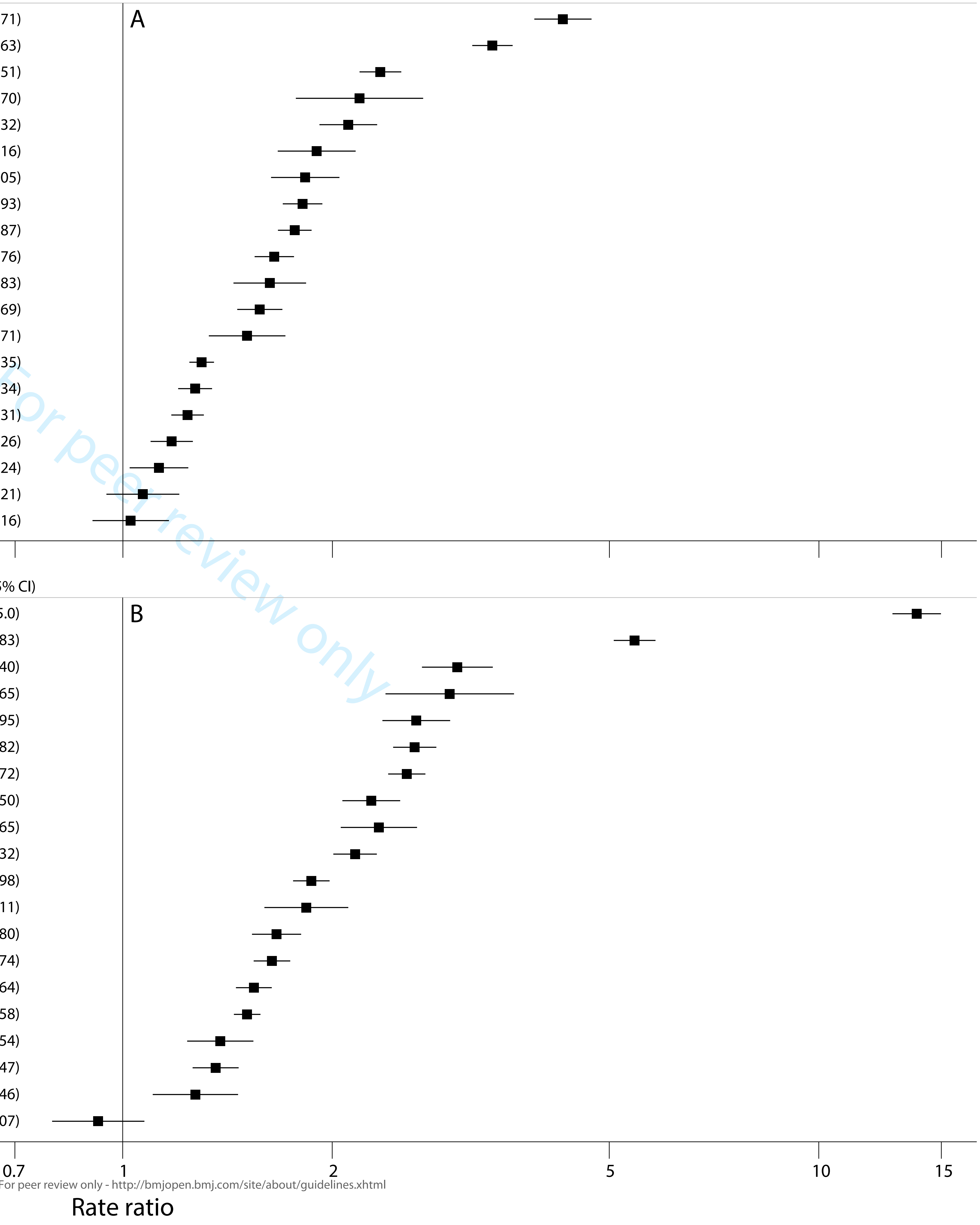

For peer review only
